# A worldwide perspective on large carnivore attacks on humans

**DOI:** 10.1371/journal.pbio.3001946

**Published:** 2023-01-31

**Authors:** Giulia Bombieri, Vincenzo Penteriani, Kamran Almasieh, Hüseyin Ambarlı, Mohammad Reza Ashrafzadeh, Chandan Surabhi Das, Nishith Dharaiya, Rafael Hoogesteijn, Almira Hoogesteijn, Dennis Ikanda, Włodzimierz Jędrzejewski, Mohammad Kaboli, Anastasia Kirilyuk, Ashish Kumar Jangid, Ravi Kumar Sharma, Hadas Kushnir, Babu Ram Lamichhane, Alireza Mohammadi, Octavio Monroy-Vilchis, Joseph M. Mukeka, Igor Nikolaev, Omar Ohrens, Craig Packer, Paolo Pedrini, Shyamala Ratnayeke, Ivan Seryodkin, Thomas Sharp, Himanshu Shekhar Palei, Tom Smith, Ashok Subedi, Fernando Tortato, Koji Yamazaki, Maria del Mar Delgado

**Affiliations:** 1 MUSE – Science Museum, Research & Collections Department, Conservation Biology Unit, Trento, Italy; 2 Department of Evolutionary Ecology, National Museum of Natural Sciences (MNCN), Spanish National Research Council (CSIC), Madrid, Spain; 3 Dept. of Nature Engineering, Agricultural Sciences and Natural Resources University of Khuzestan, Mollasani, Iran; 4 Department of Wildlife Ecology and Management, Faculty of Forestry, Düzce University, Düzce, Turkey; 5 Terrestrial Ecology Research Group, Dept. for Life Science Systems, Technical University of Munich, Freising, Germany; 6 Dept. of Fisheries and Environmental Sciences, Faculty of Natural Resources and Earth Sciences, Shahrekord University, Shahrekord, Iran; 7 Dept. of Geography, Barasat Government College, Barasat, Kolkata, West Bengal, India; 8 Wildlife and Conservation Biology Research Lab, Dept. of Life Sciences, Hemchandracharya North Gujarat University, Patan, Gujarat, India; 9 Panthera, New York, New York, United States of America; 10 Dept. of Human Ecology, Cinvestav, Merida Unit, Mérida, Mexico; 11 Tanzanian Wildlife Research Institute, Arusha, Tanzania; 12 Centro de Ecología, Instituto Venezolano de Investigaciones Científicas IVIC, Caracas, Venezuela; 13 Dept. of Environmental Science, Faculty of Natural Resources, University of Tehran, Karaj, Iran; 14 Daursky State Nature Biosphere Reserve, Zabaikalsky Krai, Onosky District, Nizhniy Tsasuchey, Russia; 15 Wildlife Institute of India, Dehradun, India; 16 HCL Foundation, HCL Technologies Hub, Noida, India; 17 United States Agency for International Development, Washington, DC, United States of America; 18 NTNC – Biodiversity Conservation Center, Sauraha, Nepal; 19 Dept. of Environmental Science and Engineering, Faculty of Natural Resources, University of Jiroft, Jiroft, Iran; 20 Universidad Autónoma Del Estado De México Toluca, México y Universidad Autónoma Metropólitana-Lerma, Lerma de Villada, México; 21 Wildlife Research and Training Institute, Naivasha, Kenya; 22 Federal Scientific Center of the East Asia Terrestrial Biodiversity FEB RAS, Vladivostoka, Vladivostok, Russia; 23 Dept. of Ecology, Evolution and Behavior, Univ. Minnesota, St. Paul, Minnesota, United States of America; 24 Aga Khan University, Arusha, Tanzania; 25 Dept. Of Biological Sciences, Sunway University, n.5 Jalan University, Selangor, Malaysia; 26 Laboratory of Animal Ecology and Conservation, Pacific Geographical Institute FEB RAS, Vladivostok, Russia; 27 Wildlife SOS – USA/India, Salt Lake City, Utah, United States of America; 28 Aranya Foundation, Bhubaneswar, Odisha, India; 29 Dept. of Plant and Wildlife Sciences, Brigham Young University, Provo, Utah, United States of America; 30 National Trust for Nature Conservation, Annapurna Conservation Area Project, Pokhara, Nepal; 31 Forest Ecology Laboratory, Dept. of Forest Science, Faculty of Regional Environmental Science, Tokyo University of Agriculture, Tokyo, Japan; 32 Biodiversity Research Institute (IMIB; CSIC-Oviedo University, Principality of Asturias), Campus Mieres, Mieres (Asturias), Spain; Princeton University, UNITED STATES

## Abstract

Large carnivores have long fascinated human societies and have profound influences on ecosystems. However, their conservation represents one of the greatest challenges of our time, particularly where attacks on humans occur. Where human recreational and/or livelihood activities overlap with large carnivore ranges, conflicts can become particularly serious. Two different scenarios are responsible for such overlap: In some regions of the world, increasing human populations lead to extended encroachment into large carnivore ranges, which are subject to increasing contraction, fragmentation, and degradation. In other regions, human and large carnivore populations are expanding, thus exacerbating conflicts, especially in those areas where these species were extirpated and are now returning. We thus face the problem of learning how to live with species that can pose serious threats to humans. We collected a total of 5,440 large carnivore (Felidae, Canidae, and Ursidae; 12 species) attacks worldwide between 1950 and 2019. The number of reported attacks increased over time, especially in lower-income countries. Most attacks (68%) resulted in human injuries, whereas 32% were fatal. Although attack scenarios varied greatly within and among species, as well as in different areas of the world, factors triggering large carnivore attacks on humans largely depend on the socioeconomic context, with people being at risk mainly during recreational activities in high-income countries and during livelihood activities in low-income countries. The specific combination of local socioeconomic and ecological factors is thus a risky mix triggering large carnivore attacks on humans, whose circumstances and frequencies cannot only be ascribed to the animal species. This also implies that effective measures to reduce large carnivore attacks must also consider the diverse local ecological and social contexts.

## Abstract 2

A worldwide study of large carnivore attacks on people analyzes and compares the main patterns and circumstances within and among species and across geographic regions, shedding light on this complex and critical type of human-wildlife conflict.

## Introduction

Human impact on the natural world is increasing: 10 million km^2^ of land is projected to be modified for human use by 2050 [1], as the human population is predicted to grow to around 9.7 billion [2]. Such an increase in the human population has made our species a dominant force on the planet, prompting geologists to define the present period as the Anthropocene [3]. With approximately 50% to 70% of Earth’s land surface currently modified for human activities [4], the expanding human footprint is causing not only the loss of habitat and biodiversity, but also alterations in animal behavior as species attempt to survive displacement and increasingly fragmented and disturbed habitats [3].

Large carnivores have long fascinated human societies, yet their persistence has emerged as one of the greatest conservation challenges of our time [5,6]. These species can pose threats to people living near them, sometimes resulting in human injury and death, especially in multiuse landscapes where human activities and large carnivore ranges overlap [7–9]. This particular aspect of human-wildlife conflict urgently demands improvement of our knowledge to successfully manage human–large carnivore coexistence under different conditions [7,10]. In many high-income regions, large carnivores are recolonizing parts of their historical ranges, mainly due to a combination of decreased persecution and the recovery of natural habitats and wild prey [11–13], creating novel scenarios of human–carnivore coexistence [13]. This phenomenon has brought populations of large carnivores closer to humans in areas where habitats are fragmented and encroached upon by towns, villages, roads, farmland, crops, and a variety of other human activities [5,14,15]. In these areas, conflicts might be especially severe since traditional prevention and coexistence adaptations have been lost and people are no longer accustomed to sharing the landscape with large predators [11,16]. In other areas of the world, large carnivore populations are declining because of expanding human populations causing relevant habitat loss, fragmentation, and degradation [17–20]. Under both scenarios, such close coexistence produces anthropogenic changes to species behavior [4,21–24] and, consequently, human–wildlife conflicts [25].

We thus face one of the most daunting challenges of our time, i.e., learning to live with large animal species that may cause serious conflicts such as attacks on humans [7,26]. Humans are not the only victims in large carnivore attacks, as attacks have extremely negative impacts, both direct and indirect [27,28], on large carnivore conservation as well. The animals involved in attacks are often killed during conflicts or removed afterwards from the population [27,29,30], which have long-term conservation consequences [31]. Moreover, although rare, attacks reinforce negative attitudes toward the species [28,32]. Reducing these incidents should thus be considered a priority to improve coexistence and, consequently, ensure human safety and the long-term conservation of large carnivore populations.

Various studies have investigated large carnivore attack patterns and correlates at different geographic scales and focusing on one or more species (see S2 Table for a full list of publications on the topic), but no study has addressed the issue on a global scale and included all species of large terrestrial carnivores. By all species of large terrestrial carnivores, we mean the 12 carnivore species included in the families Felidae, Canidae, and Ursidae for which attacks on humans have been recorded.

Here, our aim is to provide a global overview of large carnivore attacks on humans to compare patterns within and among species and across geographic regions. This perspective is crucial to understanding the roots and complexity of such conflicts, reducing their occurrence and avoiding erroneous generalizations when addressing the issue. We hypothesize that encounters with carnivores in high-income countries are mostly the result of high-risk human behaviors when deliberately entering areas inhabited by large carnivores, e.g., recreational activities. Conversely, in low-income countries, coexistence between humans and large carnivores is mostly involuntary. Here, communities and villages are often located in large carnivore habitats, or close to the edges of protected areas, where people are exposed to risky encounters with these species during daily routines and livelihood activities.

We first analyze how the number of reported attacks has varied throughout the years, and whether the long-term patterns in different regions of the world have been similar, to specifically examine differences in the circumstances of the reported attacks on humans across large carnivore species and regions of the world. We hypothesize that the annual trend, as well as the circumstances, of the reported attacks (i) differ across regions of the world and among carnivore families as a result of the local socioeconomic and ecological contexts. In particular, we expect that the number of attacks increases over time in low-income regions, where subsistence economies are still largely present and many communities still live in close contact with wildlife, including large felids.

Secondly, we focused on predatory attacks, i.e., incidents where humans were attacked with the likely purpose of being consumed [8]. We considered that an attack was predatory when [8] (1) human victims were treated as food, i.e., the victim, still alive, was dragged by the carnivore from the attack point to a more concealed location (e.g., bushes or within a forest patch); (2) the body was hidden and covered with leaves and soil; (3) the victim was partially consumed after death; and/or (4) a large carnivore was found near the body. However, we discarded those incidents where there was no evidence that the human body had been consumed immediately after the kill. Finding a partially eaten body days after the disappearance of a person could have represented a scavenging event following a death not directly linked with a large carnivore attack. Whereas a considerable number of attacks are the result of defensive reactions by animals, and may be avoided by adopting preventive measures and appropriate behaviors when entering large carnivore habitat [7], predatory attacks are the most dangerous because the intention of the predator is to kill for food and may be more difficult to deter [8]. Specifically, we expect that (ii) predatory attacks are mainly typical of felid and canid species, which are generally strictly carnivorous [33–35], while ursids mostly attack for defensive reasons [8,16], and (iii) death outcomes depend on the species, the attack motivation, and the region. Furthermore, (iv) children and solitary people are the main targets of these attacks, as occurs in North America, where predatory attacks mainly target the weakest and smallest individuals [8]. Low-income regions are also likely to reflect this pattern because children frequently participate in work/livelihood activities.

## Methods

### Collection of attack reports

We collected information on reported attacks on humans by all large carnivore mammal species that occurred worldwide between 1950 and 2019 (more details on attack cases and the published literature collected and analyzed is available in S1 and S2 Tables). We considered as attacks only those interactions that resulted in physical injury or death. Attack records were collected from personal datasets of the coauthors, published literature, PhD/MS theses, webpages, and news reports (a list of the main published research and reports on the topic is provided in S2 Table). We searched for the abovementioned sources using the search engines Google and Google Scholar. To complete the dataset, in addition to these sources, we also carried out a systematic search of news articles on Google for each country/region on an annual basis, using the combination of the following terms: “common species name” or “scientific species name” + “attack” or “attack” + “human.” Because some reported attacks appeared repeatedly during the search, due to the use of multiple sources, we cross-checked information such as attack location, date, and sex/age of the people involved to avoid duplicate records in the dataset (additional information on the data collection method is available in [7,36]). For brown bears *Ursus arctos*, we used information already published in a recent scientific paper [36]. We excluded confirmed and suspected cases of attacks by rabid animals because the behavior of infected individuals is typically more aggressive and thus far from natural animal behavior [37]. For each attack, we recorded the following information: date; species involved; location of the attack; time of day, classified into three categories, i.e., twilight, day, and night; sex and age (subadult or adult) of the animal; sex and age of the victim, where age was classified into two categories (child, <13 years old; adult, ≥13 years old); human party composition, simplified into four categories: (a) adult alone, (b) child alone, (c) child in a group, and (d) adult in a group; and result of the attack, i.e., injury or death. Where possible, we also recorded the main human activities at the time of the attack, which were grouped into the following macro categories: (1) work/livelihood activities, e.g., farming, grazing livestock, collecting forest products, fishing, going to school; (2) recreational activities, e.g., hiking, camping, playing, dog-walking, fishing for sport; (3) hunting/poaching (these two categories were grouped simply because they both imply similar human behavior like moving silently in the forest with a weapon); and (4) children playing. Attack scenario, i.e., the main factor that could have triggered the attack, was also recorded. We defined 7 different scenarios: (a) defensive reaction by a female with offspring; (b) involuntary sudden encounter, i.e., the animal and the person surprised each other at a close distance or the person involuntarily disturbed the animal while sleeping or denning; (c) food-related attack, e.g., the animal defended a carcass, the predator was surprised while attacking livestock, or it was feeding on anthropogenic food (e.g., crop, trash) at the time of the attack; and (d) predatory/unprovoked attack, i.e., the animal deliberately attacked and/or killed a human with the presumed purpose of consuming it [38], or without any apparent provocation by the person. We decided to group predatory and unprovoked scenarios since we did not always have enough details to consider it as predatory, although it was certainly deliberate. In any case, reported attacks classified as unprovoked were few (*n =* 9). In this category, we also included investigative attacks, i.e., when the animal intentionally attacks a person with the presumed purpose of testing or investigating them as potential prey, a scenario especially described for canids [39,40]. Usually, adults are involved in these attacks and the animal does not press the attack but readily flees after the person reacts [40]. Other scenarios included (e) the animal attacked after being wounded or trapped (e.g., during hunting/poaching or research/monitoring activities); (f) dog-related scenarios, i.e., one or more dogs (*Canis lupus familiaris*) were present at the moment of the attack and likely caused the attack; and (g) the animal attacked after being intentionally approached/provoked/chased by people. We also collected other information that could help us to better interpret the attack circumstances (see S1 Data).

We acknowledge that our dataset does not include the totality of attack cases that actually occurred worldwide, and thus it represents a subsample of cases. Indeed, although the effort put into collecting cases was equal for each species and region, many cases, especially those involving species such as lions *Panthera leo*, leopards *Panthera pardus*, and tigers *Panthera tigris*, are missing from our dataset. Importantly, even though some information presented in S1 Data was not analyzed in the context of the present study, we considered it important to make it available because this unique information could be further analyzed in future studies.

### Information related to the socioeconomic situation of the countries where attacks were collected

For each country in which attack cases were collected, we extracted information from the World Bank Group (https://www.worldbank.org/) concerning its socioeconomic situation, the degradation and loss of habitat, climate change and ecosystem breakdown, and the expansion of agricultural and its rural population (S1 Table). Specifically, we collected the following information: (1) *Environment – land use*: agricultural land, arable land, and forest land (each one as a % of land area); (2) *Environment – emissions*: CO_2_ emissions (metric tons per capita); (3) *Environment – density and urbanization*: rural population (% of the total population); (4) *Infrastructure – communications*: individuals using the internet (% of the population); and (5) *Economy Policy and Debt*: gross national income (GNI) per capita in US dollars in a year. In addition, we classified the economy of each country into the following four categories, following the World Bank Group classification based on GNI per capita in US dollars in a year [41]: (1) low income (≤$1,005); (2) lower middle income ($1,006 to $3,975); (3) upper middle income ($3,976 to $12,275); and (4) high income (>$12,275). We chose the year 2010 as a reference year to extract income values and classifications because it represents an intermediate point between past and present economic situations. It was not our intention to characterize the exact locations where attacks occurred, but to relate the long-term patterns and circumstances of the reported attacks in different regions with global information at the country scale.

### Statistical analyses

To quantify how the number of reported attacks has varied throughout the years, we fitted a generalized additive mixed model (GAMM), treating the number of attacks recorded in each country by the different large carnivore species as a response variable, and the year as a smoothing variable using the default thin-plate regression spline in the GAMM4 package in R [42,43]. We removed the attacks recorded before 1970 because there were very few cases and a lack of information at the country scale before that year. Because our data were overdispersed, we fitted the GAMM using a Negative Binomial distribution instead of a Poisson distribution. Furthermore, as the number of attacks was count data with no zeros, we modeled the response variable number of attacks-1 as a simple way to technically consider it as a zero-truncated Negative Binomial regression. To measure whether the long-term patterns in the number of attacks have been similar in different regions of the world, we further included as smoothing variables the interactions of year with the % of agricultural, arable, and forest land areas, the % of the rural population, CO_2_ emissions, and the GNI as both a continuous and a categorical variable. When adding the nonlinear effects, we checked the effective degrees of freedom (EDF) of the variables. All variables showed an EDF > 2 and were therefore retained in the model as nonlinear predictors [42]. To exclude collinearity among the explanatory variables, we calculated the variance inflation factor (VIF; [44]). We excluded the % of arable land, the % of the rural population, and the GNI (as a continuous variable) because their thresholds were >2. Furthermore, to account for any potential bias due to differences in the number of reported attacks collected among years, species, and countries, we included the country ID, the species ID, and the year ID as random intercept factors. By doing so, we simultaneously accounted for any other potential influential factors varying with site, species or year that could have otherwise been overlooked. Finally, to account for the fact that the number of reported cases in each country might have increased from the premodern technology to the modern technology period, we included internet coverage nationwide (i.e., the % of individuals using the internet each year in each country) as an offset in the model.

Once we generated the sets of competing models, we employed the Akaike information criterion (AIC), using the value of ΔAIC < 2 as the criterion for selecting the most parsimonious model. Models were finally evaluated by checking diagnostic plots. All analyses were performed using R 4.0.4. [45].

## Results and discussion

**Fig 1:**
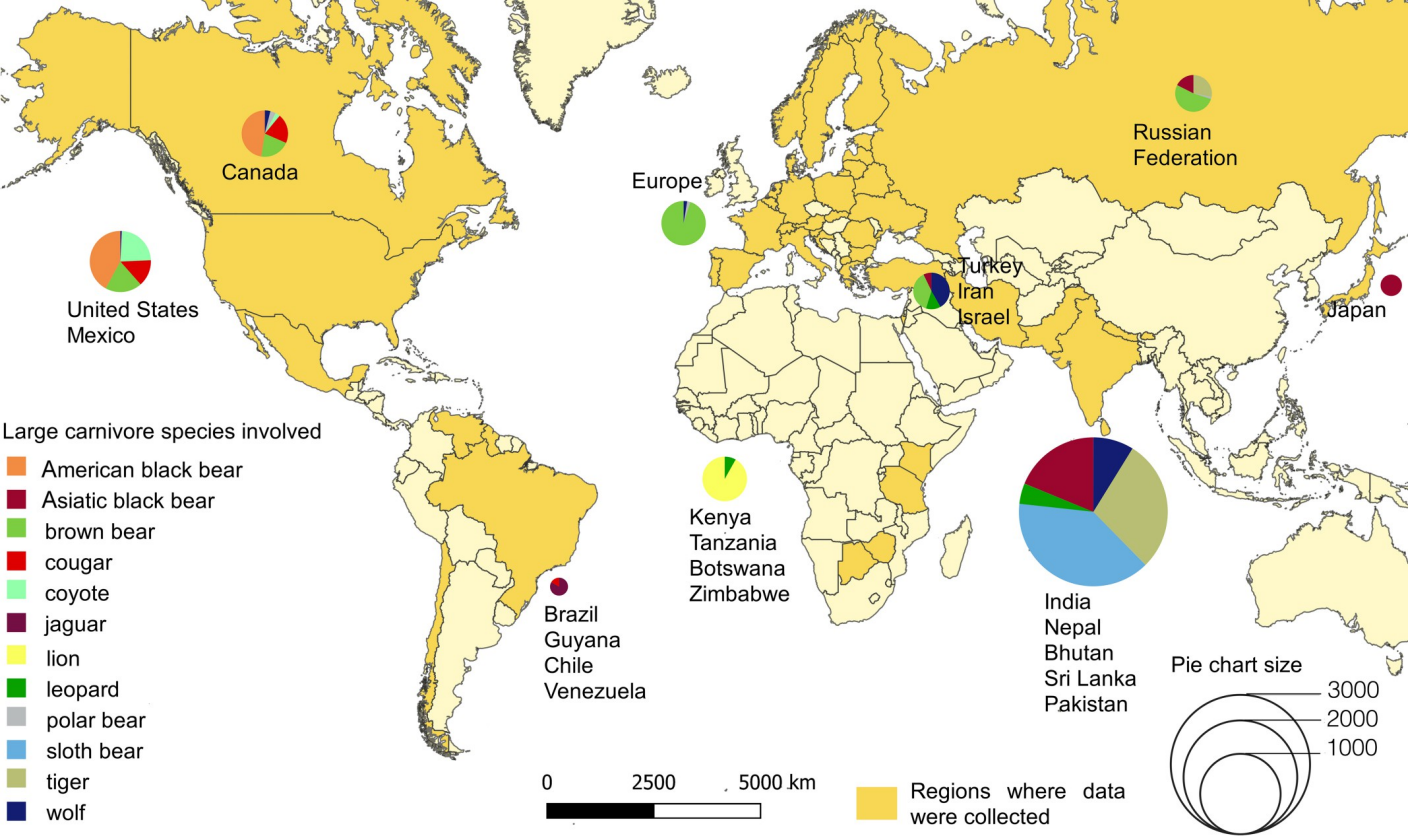
Spatial distribution of large carnivore attacks on humans collected between 1950 and 2019. We collected a total of 5,440 attack cases worldwide: 1,337 involved sloth bears *Melursus ursinus*, 1,047 tigers *Panthera tigris*, 765 Asiatic black bears *Ursus thibetanus*, 664 brown bears *Ursus arctos*, 414 wolves *Canis lupus*, 403 American black bears *Ursus americanus*, 282 lions *Panthera leo*, 205 leopards *Panthera pardus*, 140 coyotes *Canis latrans*, 135 cougars *Puma concolor*, 25 jaguars *Panthera onca*, and 23 polar bears *Ursus maritimus*. The maps were produced in QGIS, and the base shapefile layer of world countries was downloaded from Natural Earth (https://www.naturalearthdata.com/downloads/10m-cultural-vectors/10m-admin-0-countries/) and do not require credit because of public domain. The data underlying this Figure can be found in S2 Data.

Our search resulted in a total of 5,440 large carnivore attacks on humans worldwide between 1950 and 2019 (Fig 1 and S1 Table).

**Fig 2:**
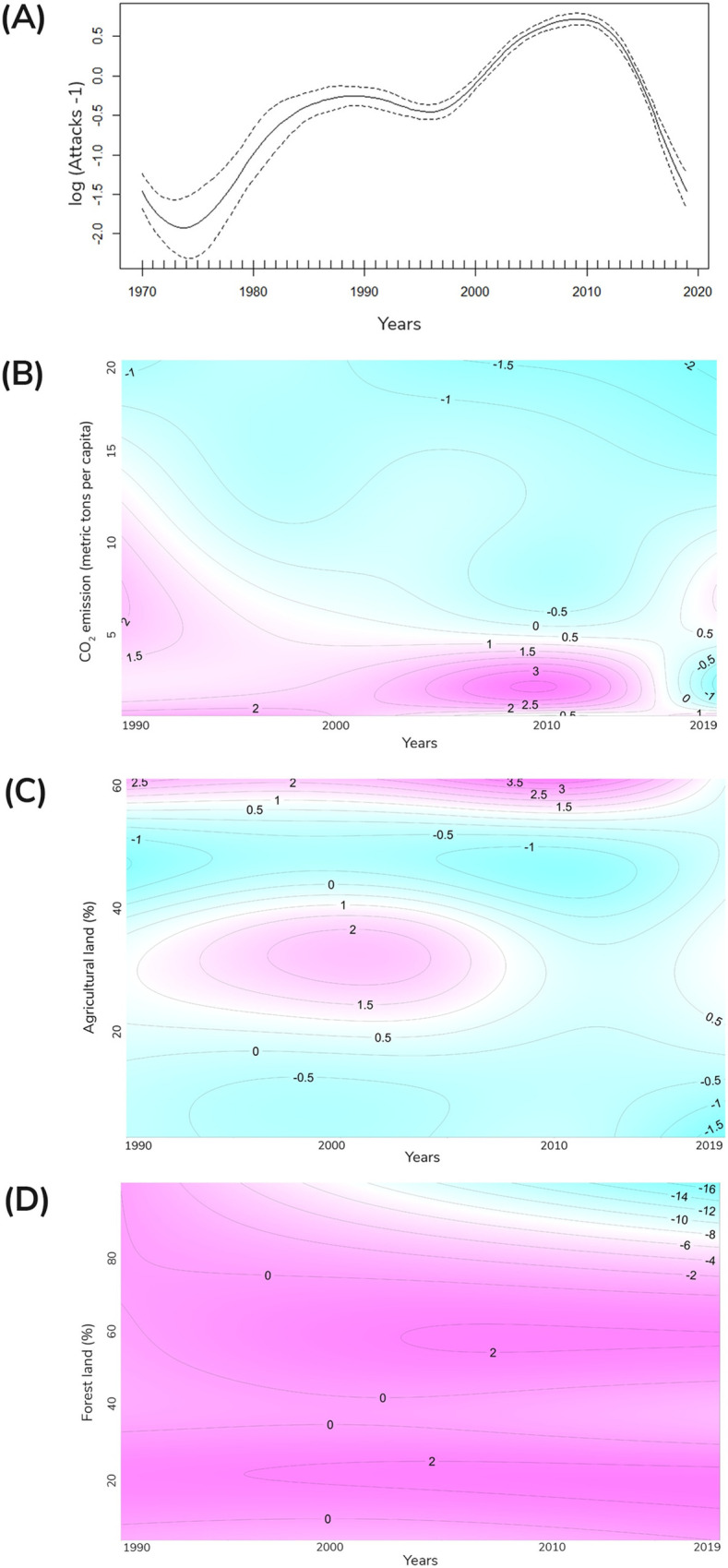
Temporal trends in large carnivore attacks on humans in different regions of the world. (**A**) The number of attacks shows a nonlinear increase over the years, as evidenced by fitting the general additive mixed model of the number of attacks-1 as a function of the smoothing factor “year.” In particular, the number of attacks has increased in countries with (**B**) low CO_2_ emissions and (**C**) a high proportion of agricultural land area. In countries with (**D**) large forest coverage, the number of attacks in the last several years has decreased. Panels B-D are counterplots representing, respectively, the effect of the interaction between year and CO_2_ emissions, % of agricultural land area, and % of forest land area on number of attacks-1 from a generalized additive mixed model. The axes represent the values of the predictor variables, and the interior is a topographic map of the predicted values. The pink colors represent larger predictions and the blue ones smaller predictions. The data underlying this Figure can be found in S2 Data.

We found that the number of reported attacks has nonlinearly increased over the years (Fig 2A), especially in lower-income countries that are generally characterized by low CO_2_ emissions (Fig 2B) and a high proportion of agricultural land (Fig 2C). Furthermore, while in the early 1990s the number of attacks was similar in countries with different forest coverage (Fig 2D), the number of attacks has significantly decreased over the years in those countries with a high proportion of forest.

Most attacks (68%, *n =* 3,459) resulted in human injuries, whereas 32% (*n* = 1,630) were fatal. Yet, attack scenarios vary greatly within and among species, as well as in different areas of the world. An example of how extreme the difference in these scenarios can be depending on the area is well represented by the different patterns of wolf attacks between the Holarctic and India. In Europe and North America, very few wolf attacks occurred (*n* = 25) in the 70 years covered by our study, and in almost all predatory/unprovoked attacks (15 out of 18), the animal was food conditioned. In this part of the world, wolves usually do not pose a threat to people unless they are provoked, or their behavior has been altered because of food conditioning. But the situation changes drastically in Indian regions, where predatory wolf attacks were more frequent (at least 300 cases) during the same time span. Similarly, tiger attacks were mostly predatory in the Sundarbans mangrove area (Bay of Bengal) and leopard attacks were mostly predatory in the Kashmir region, whereas tiger attacks in Russia and leopard attacks in other areas of India were mostly defensive, i.e., defensive reactions to involuntary encounters with people or to defend offspring (see also Fig A in S1 File).

Large felids such as tigers and lions caused more deaths in general, with 65% of felid attacks being fatal, followed by canids (49%) and ursids (9%; Fig B in S1 File). This is likely due to the fact that felids and canids were more frequently involved in predatory events, and both are well adapted to effectively prey upon large animals [46]. Instead, ursids were mainly involved in involuntary sudden encounters (45%), defensive reactions by females with cubs (18%), or food-related interactions (16%), such as bears defending a carcass, or being surprised while attacking livestock or feeding on anthropogenic food. Mortality rates also vary as a result of such factors as attack motivation, characteristics of the person involved, e.g., age and party composition (Fig C-E in S1 File), and geographic region (Fig F and G in S1 File), again suggesting that death outcomes are not solely dependent on the carnivore species. Actually, most fatal attacks (85%) occurred in lower-income countries (low- and lower middle-income categories; Fig F in S1 File), likely because tigers and lions, whose attacks were mainly predatory, are almost exclusively present in these regions (S1 File): 91% of the total deaths recorded were the result of predatory attacks. Furthermore, the fact that victim rescue and hospitalization procedures are slower than in higher-income regions, or sometimes completely lacking [29,47,48], likely contributes to reducing the survival chances of the human victim.

**Fig 3:**
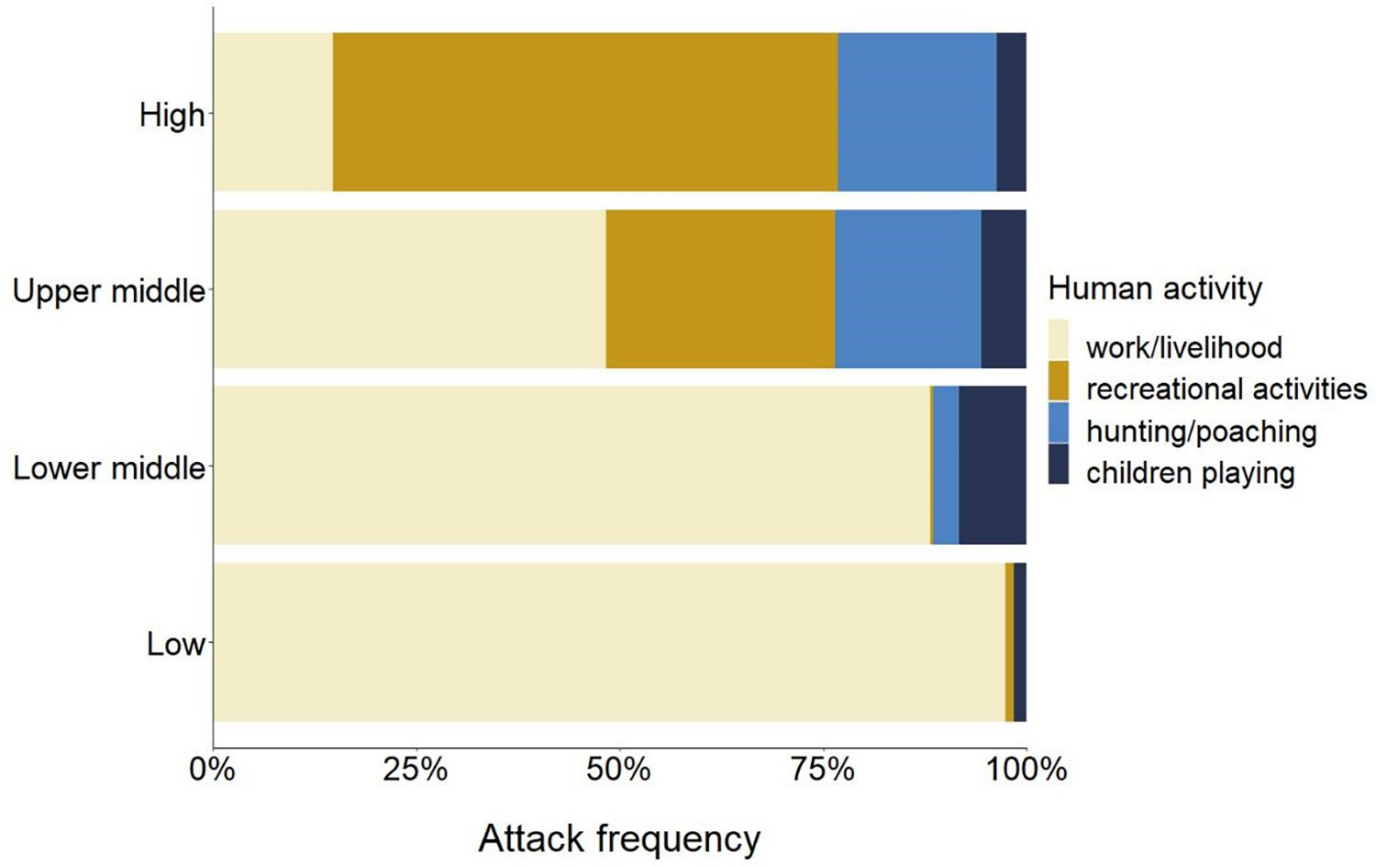
Attack circumstances clearly differ by regional income levels on a global scale. In higher-income regions, attacks mainly involved people engaged in recreational activities, whereas in lower-income countries, attacks primarily involved people carrying out work or livelihood activities. The y-axis shows income levels of the countries where attacks occurred: (1) low income (≤$1,005); (2) lower middle income ($1,006–3,975); (3) upper middle income ($3,976–12,275); and (4) high income (>$12,275) [41]. The data underlying this Figure can be found in S2 Data.

Human activity at the time of the attack also varies by region. In countries with higher incomes, people engaged in recreational activities comprised the majority (48%, *n =* 604) of attack victims, whereas in lower-income countries, most people (89%, *n* = 2,230) were attacked during work or livelihood activities, such as farming, grazing livestock, fishing, and collecting forest products (Fig 3). These results highlight the clear difference in circumstances leading to attacks in low- and high-income countries, and the special challenges faced by low-income nations. In high-income regions, such as most North American states, human activities and large carnivores are frequently spatially separated, and most people involved in conflicts are those (a) visiting natural areas for recreational purposes [7,9,36,49]; or (b) living in urban and nearby landscapes, where potentially dangerous species may approach houses or inner-city parks, e.g., because they are involuntary attracted by anthropogenic food, or voluntarily fed [9,50]. In contrast, in lower-income countries, a greater portion of the human population live and work in rural areas where human and large carnivore habitat and activities overlap [18,40,51] (see also S1 File and S1 Table for more details on attack circumstances per species and region).

**Fig 4:**
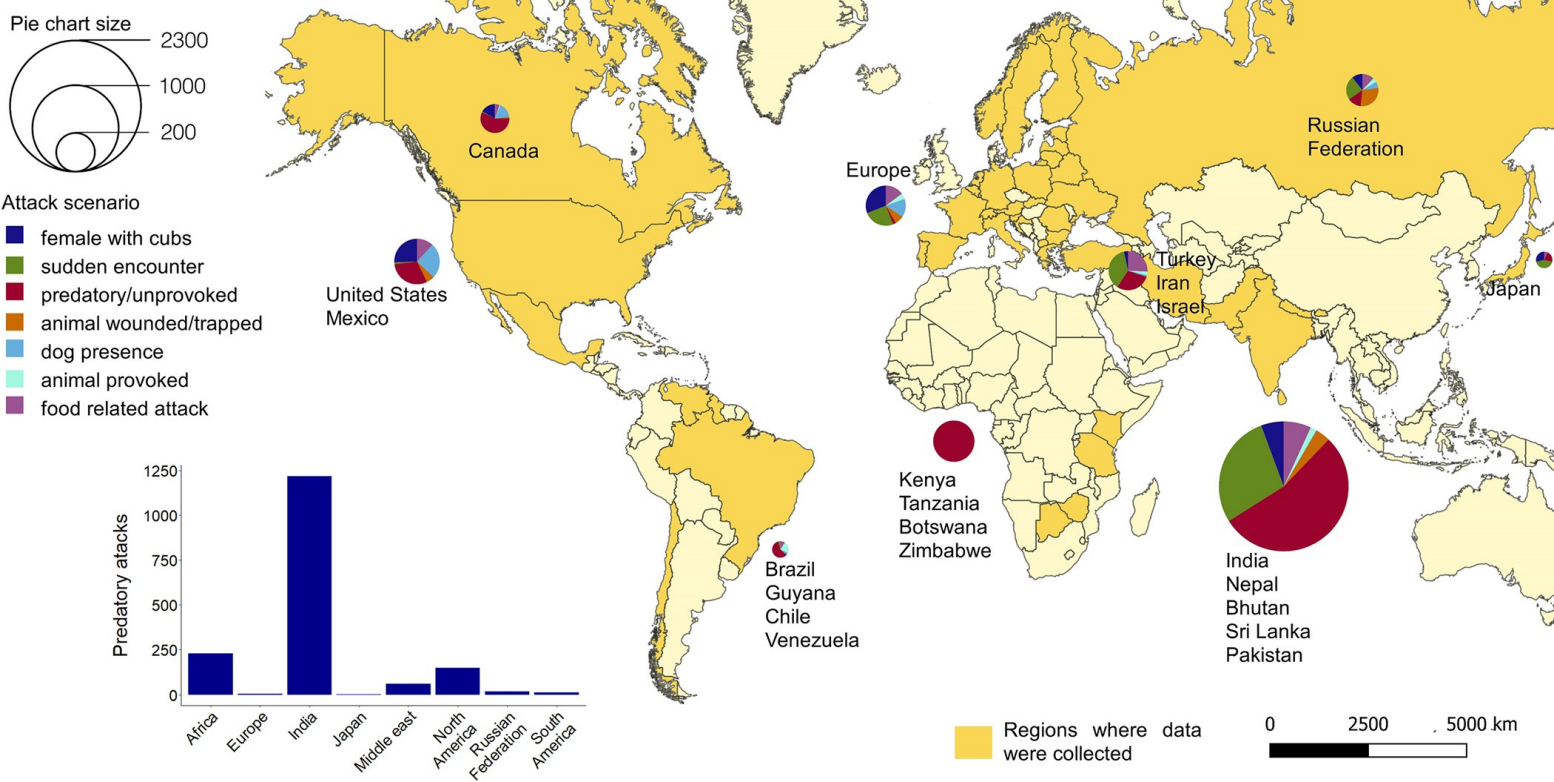
Global spatial distribution (1950–2019) of the main large carnivore attack scenarios. Predatory attacks are especially concentrated in India and Africa, where felids and canids are primarily involved. The maps were produced in QGIS, and the base shapefile layer of world countries was downloaded from Natural Earth (https://www.naturalearthdata.com/downloads/10m-cultural-vectors/10m-admin-0-countries/) and do not require credit because of public domain. The data underlying this Figure can be found in S2 Data.

Of the total cases collected globally for which the scenario was known (*n =* 3,480), 1,696 (49%) cases were classified as predatory. Predatory attacks, which mostly occurred during the night and independent of the season (but see Fig A and B in S2 File for more details), mainly involved felids (93%) and canids (88%; more details on predatory events in S2 File). Indeed, most predatory attacks occurred in low-income regions, especially India (72%) and south-eastern Africa (14%), where tigers, leopards, lions, and wolves were mainly involved (Fig 4). However, in contrast to what generally occurs in North America [8,9], the victims were mostly adults (70%) in India and south-eastern Africa (Fig C in S2 File). The age of the victim varied depending on the species and the local context. For most encounters, the victims of cougars, coyotes, wolves, and leopards were mainly children (range: 25% to 95%, mean = 54.4%; see also Fig C in S2 File), which aligns with both the abovementioned literature and our expectations. Conversely, most predatory attacks by large felids in Asia and Africa were on adults (Fic C in S2 File). One possible explanation is that in lower-income countries, especially in those areas where felids have specialized in preying on humans, adults are more likely to enter carnivore habitat while working or carrying out other livelihood activities, whereas children likely remain near houses and villages.

Too often generalizations are made when speaking of the risk posed by a given species, yet it is evident from our study that large carnivore attacks on humans represent a complex phenomenon. Our results highlight that the circumstances and frequencies of attacks are not determined by the species alone, but by the local context as a whole, with its specific combination of socioeconomic and ecological factors. This implies that several factors including the species and both the local socioeconomic and ecological context need to be evaluated when planning measures to reduce the occurrence of such attacks. Specifically, two main scenarios emerge from our global analysis. The first one is represented by high-income regions. Here, people enter or live near large carnivore areas mostly by choice, and attacks may be reduced by implementing information campaigns, targeting both visitors and people residing in large carnivore areas, about the risks and the most appropriate behaviors to prevent dangerous encounters. In such contexts, attacks mainly result from high-risk human behaviors (e.g., moving alone and being silent, leaving children unattended, or walking an unleashed dog when in large carnivore areas, intentionally or unintentionally feeding predators, thus attracting them to inhabited areas) and could be reduced by improving public awareness. Further preventive measures such as proper garbage management, both in inhabited areas frequented by carnivores and in natural areas frequented by tourists, can help prevent predators from approaching people and settlements [9,36,52]. The second scenario is that of low-income regions. Here, coexistence is mostly involuntary, and conflict reduction would require not only informing residents, but also changes in the socioeconomic context. As highlighted by the existing literature, forest fragmentation, habitat heterogeneity, e.g., a mix of natural and anthropogenic landscapes [17], and the lack of natural prey [27,30,53] represent major drivers of large carnivore attacks on humans, especially predatory attempts. In particular, attack hot spots have been identified near forest edges where human and carnivore activities overlap and human density is high [17,54]. In such contexts, domestic livestock is often available to predators, leading to increased attractiveness of areas exploited by humans to these animals [27]. Preventive measures, intended to separate humans and their livelihood activities from predators and to give predators more safe space and natural prey to rely on to reduce conflicts, include zoning and expanding protected areas, which often entails the relocation of human communities, improving and restoring habitats and habitat connectivity, and increasing natural prey availability through better harvesting and decreased competition with livestock [17,27,54]. However, if human populations continue to increase at current rates, the idea of providing predators more space without worsening habitat encroachment and affecting local communities will be challenging. Addressing the issue must thus be a priority to improve coexistence and the long-term conservation of large predator populations.

## Supporting information

S1 File Main attack patterns. Main characteristics and patterns of large carnivore attacks on humans documented in our study.
(PDF)

S2 File Focus on predatory attacks. Characteristics of large carnivore predatory attacks on humans documented in our study.
(PDF)

S1 Table Details of cases collected. Details of large carnivore attacks on humans collected between 1950 and 2019 on a worldwide scale.
(PDF)

S2 Table Main published literature. Main published literature collected and analyzed regarding large carnivore attacks, organized by species.
(PDF)

S1 Data Complete dataset of large carnivore attacks on humans documented between 1950 and 2019. (XLSX)

S2 Data Numeric data underlying findings and figures. (XLSX)

## Abbreviations

AIC: Akaike information criterion
EDF: effective degrees of freedom
GAMM: generalized additive mixed model
GNI: gross national income
VIF: variance inflation factor
