# Supplementary material for: A worldwide perspective on large carnivore attacks on humans
Source: PLoS Biol. 2023 Jan 31;21(1):e3001946. doi: 10.1371/journal.pbio.3001946 (PMC9888692; doi:10.1371/journal.pbio.3001946)
Supplement: S1 File — Main characteristics and patterns of large carnivore attacks on humans documented in our study. (PDF) [file pbio.3001946.s001.pdf]

S1 File. Main characteristics and patterns of large carnivore attacks on humans documented in our study.

Main attack circumstances by species and species family

Felidae 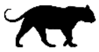

Almost all of the attacks by felid species were the result of predatory attempts on humans (93%, n = 1227, Fig A). According to our expectations, this family caused the highest mortality rates, with attacks ending with the death of the person in 65% of the cases (n = 1091; Fig B). Contrary to our expectations, most people involved in attacks were adults (88%, n = 1258), whereas children were rarely involved. We found considerable differences among species and geographic regions.

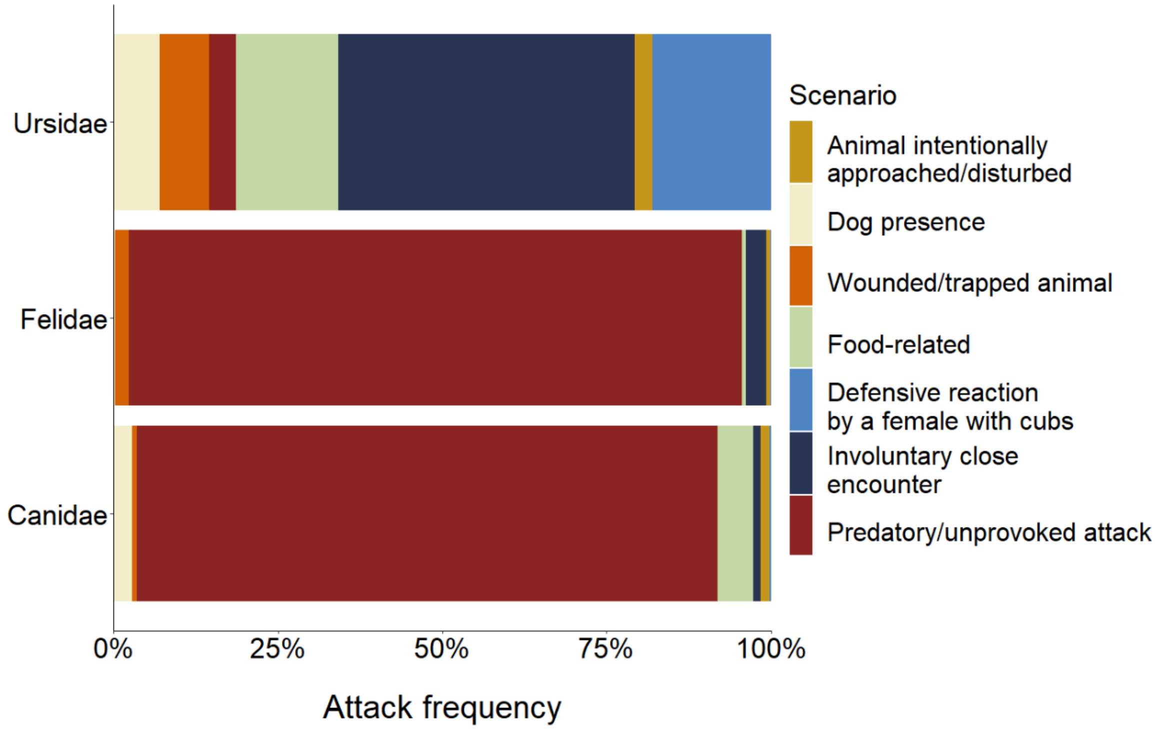

Fig A. Main attack scenario by species family. The data underlying this Figure can be found in S2 Data.

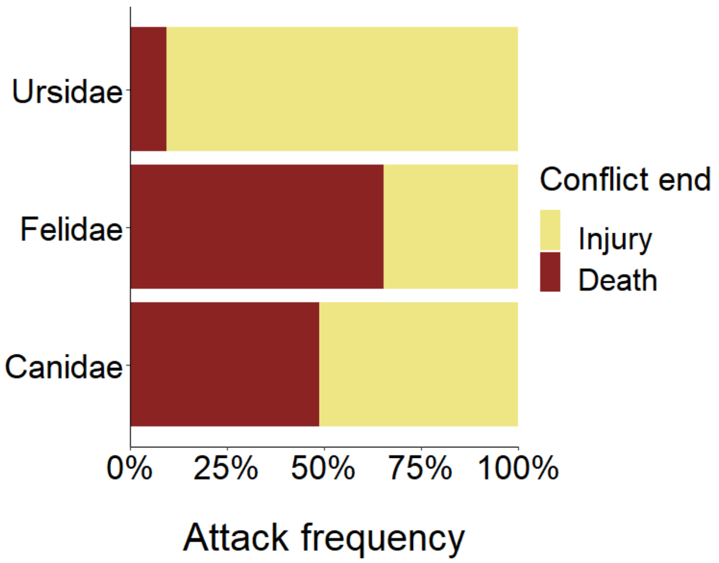

Fig B. Conflict end by species family. The data underlying this Figure can be found in S2 Data.

Cougar (*Puma concolor*). Most attacks were predatory (93% out of 61 cases where the scenario was known; Fig C). At the moment of the attack, most people were involved in sport activities or hiking (37%), playing (14%), camping (13%), walking (12%) or other less frequent activities. People that were attacked died in 12% of the cases (n = 19 out of 156 cases for which this information was available; Fig D) and half of the victims were children (54%, n = 75; Fig E). We recorded 127 cases from North America, and only a few (n = 8) in Mexico, Chile and Brazil. The limited number of attacks in the latter area likely reflects the real number of attacks, suggesting that fewer negative interactions of this kind occur compared to North America.

Jaguar (*Panthera onca*). Almost half (47%, n = 8 of 17 cases for which this information was known) of the attacks were predatory, followed by attacks due to the animal being intentionally provoked (29%, n = 5), defending a carcass (12%, n = 2) and other less frequent scenarios (Fig C). Attacks by this species ended with the death of the person in 29% of the cases (n = 7; Fig D). Most of the victims were adults (88%, n = 21; Fig E). Thirty-nine percent of people were attacked while hunting or poaching, whereas 13% of the people were fishing (as a livelihood activity), 13% were farming and the rest were involved in other activities.

Leopard (*Panthera pardus*). Half of the cases occurred in India (n = 80). More than half (63%, n = 50) of these attacks were predatory, followed by attacks due to the animal being involuntarily surprised at close quarters (35%, n = 28; Fig C). Attacks were fatal in 27% of the cases (n = 44; Fig D). Most people involved were adults (67%, n= 72; Fig E). Thirty-five percent of the people involved were grazing livestock at the time of the attack, 18% were collecting forest products and 13% were farming. Attack

scenarios vary among regions: leopards mainly attacked with predatory intent (96%) in the Kashmir Valley (India, *Panthera pardus fusca*), whereas most attacks were defensive in other regions of India (100%) and in Iran (85%) (*Panthera pardus saxicolor;* Fig 4 and S1 Table). Previous studies on the subspecies *Panthera pardus fusca* also found that predatory attacks occur in some areas, whereas in other regions of India attacks are mainly defensive reactions to involuntary encounters with people [1].

Lion (Panthera leo). Frequencies of attack scenarios for this species are only known for Tanzania and Zimbabwe (n = 228). All these cases were the result of a predatory attempt on humans (Fig C). In Tanzania, however, only predatory cases in two study areas were reported, although other scenarios are known to occur (e.g., attacks on humans while hunting lions). Exact frequencies were unknown for attacks collected in Kenya; however, the main scenarios reported here were predatory, involuntary sudden encounters and animals attacking after being intentionally provoked (J. Mukeka pers. comm.). Fifty-nine percent of the cases ended with the death of the person involved (n = 165; Fig D). Most victims were adults (76%, n = 176; Fig E). Almost half of the people involved were engaged in various activities at home or just outside or in the surrounding property (46%), 24% were walking, and 12% were relieving themselves or bathing outside (12%). Almost all lion attacks recorded were predatory [2,3], whereas we found almost no non-predatory cases in the published literature and available reports. However, it is true that other kinds of scenarios are known (J. Mukeka and H. Kushnir unpublished data). It is thus possible that, as the frequencies of predation on humans by this species are much higher compared to other scenarios [4] and predatory attacks are more lethal, this type of attack is more likely to be reported and analysed than other kinds of attacks. Treves and Treves [2], for example, reported that in the Ugandan Game Department archives some of the reported attacks were due to people trying to defend livestock from lion attacks. Also in Tanzania, despite having different attack scenarios, including provoked and defensive attacks, most attacks are predatory and this type of scenario is the one that has been mostly explored by researchers (e.g., [4–6]).

Tiger (Panthera tigris). Attack scenarios are known for attacks that occurred in India and the Russian Federation (n = 923) only. Almost all (95%, n = 879) cases recorded were predatory (Fig C). This species caused the highest mortality rates (82%, n = 856; Fig D). All cases involved adult people (100%, n = 925; Fig E). Almost half of the people were fishing or collecting crabs (52%) and the other half were collecting forest products (41%) when the attack occurred. All attacks collected in India come from the Sundarbans region, which is a small portion of India where local communities live inside the forest and rely on its resources for a living. Attacks also occurred on the Bangladeshi side of the Sundarbans; however, we could not collect enough details to include such cases in this study. The

Sundarbans probably represents a unique situation in the country, whose circumstances likely differ from other regions. Indeed, in this area, all reported cases were predatory, which is the reason for their historical reputation as "man-eaters" in that region. However, this scenario cannot be generalized to other parts of India, where social and ecological conditions are different. In the Russian Far East, conversely, only 19% of the attacks were predatory, whereas the rest were the result of an animal being wounded or surprised at close quarters. Similar results were found in previous studies that analysed local situations [7–10]. Previous literature also seems to suggest that, for various reasons such as the occupation of degraded habitats and the lack of natural prey[11], some felid species have specialized in preying on humans in some areas [7,11,12]. Nevertheless, this behaviour is likely limited to local situations and most of the time to only few individuals of a population [12] and cannot be generalized to other areas where the species is present.

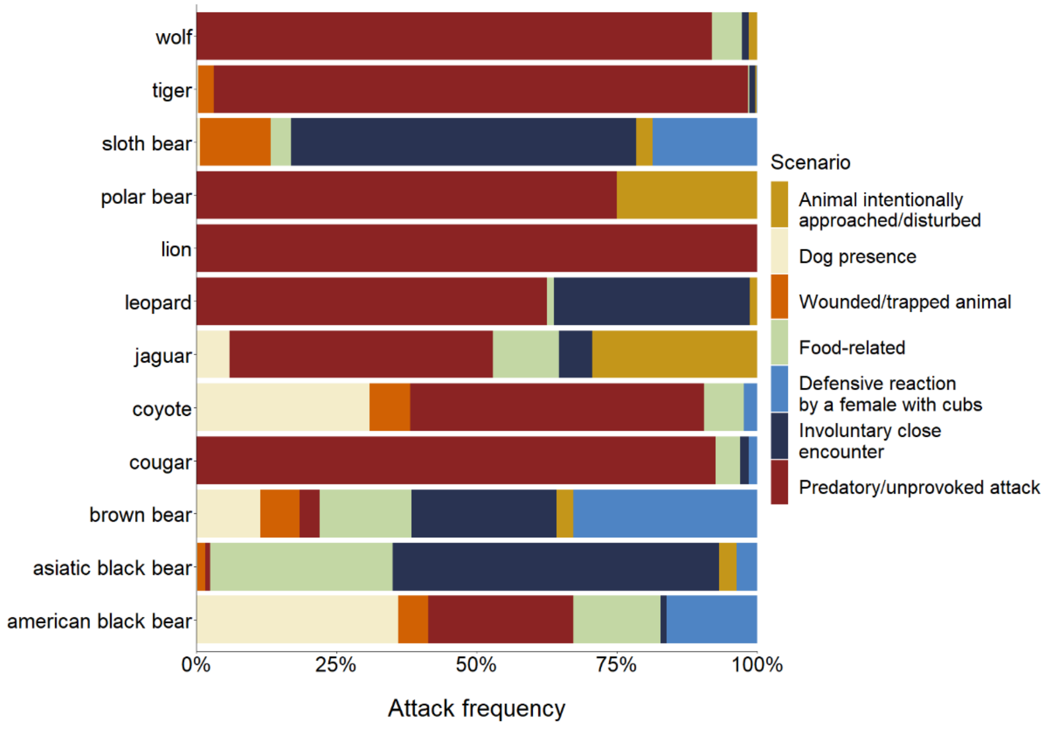

Fig C. Attack scenario per species. The data underlying this Figure can be found in S2 Data.

Ursidae 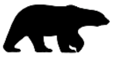

Half of the attacks by ursid species (45%, n = 772) were the result of an involuntary and sudden encounter at close distance. Other less frequent scenarios were defensive attacks by females with cubs (18%, n = 308) and food-related attacks (16%, n = 266; Fig A). Attacks ended with the death of the person in 9% of the cases (n = 269) and mainly involved adult humans (91%, n = 2277). Similar to other carnivore families, human activities at the time of the attack mainly varied depending on the region and local situation rather than on the species or family of carnivore involved (Fig 3).

American black bear (Ursus americanus). The most frequent scenarios were related to dog presence (36%, n = 67), predatory attacks (26%, n = 48), defensive reactions by females with cubs (16%, n = 30) and food-related attacks (16%, n = 29; Fig C). People died in 14% of the cases (n = 55), and adults were mostly involved (85%, n = 169; Fig D and E). Most people were camping (25%), carrying out various activities at or outside their homes (24%) or were engaged in sport activities or hiking (17%) when the attack occurred.

Brown bear (Ursus arctos). Most attacks were the result of defensive reactions by females with cubs (47%, n = 137), followed by involuntary sudden encounters (20%, n = 59), dog presence (17%, n = 48), or bears attacking after being shot or trapped (10%, n = 30). Only 5% of the attacks were predatory (n = 15; Fig C). People involved died in 14% of the cases (n = 95), and nearly all attacks involved adults (99%; Fig D and E). People were mainly engaged in recreational activities (50%, n = 279), outdoor work activities (28%, n = 158) or hunting (22%, n = 123) when the attack occurred. We found differences in attack circumstances and scenarios across different regions of their distribution range, as highlighted in previous studies[13–15].

Asiatic black bear (Ursus thibetanus). The most prevalent scenario was an involuntary sudden encounter between a bear and a person at close distance (58%, n = 256), followed by food-related causes (33%, n = 143, mostly where the bear was surprised while feeding on crops) and other less frequent motivations (Fig C). Eight percent (n = 47) of the attacks ended with the death of the person (Fig D and E), with adults being mainly involved (95%, n = 546). Farmers tending to crop fields or orchards were involved in half of the attacks (54%), followed by people collecting forest products (11%) and taking care of livestock (10%).

Polar bear (Ursus maritimus). Predation was the most common motivation behind the attacks by this species (75%, n = 3 of 4 attacks for which the scenario was known; Fig C). All attacks involved adult people (n = 26) and half of the cases were fatal (50%, n = 13; Fig D and E). Almost half (48%) of the people were camping when the attack occurred, and 38% were walking.

Sloth bear (Melursus ursinus). More than half of the cases (62%, n = 403) were the result of an involuntary sudden encounter. Another 19% (n = 122) of the cases were defensive attacks by a female bear with cubs. In 13% of the cases (n = 83) the bear had been wounded or trapped just before the attack (Fig C). It is important to note that, for this species, sometimes sudden encounters involved females with cubs, but this information was not recorded. This means that, probably, the real proportion of defensive attacks by females with cubs is likely higher than what has been reported in the database, and that the two scenarios are sometimes non-exclusive. Adult people were involved in 92% (n = 976) of the cases, and 5% (n = 59) of the cases ended with the death of the person (Fig D and E). In 22% of the cases, people were collecting forest products when the attack occurred, in 20% of the cases farmers were involved, in 19% people were attacked while relieving themselves or bathing outside, and in 10% of the cases people were taking care of livestock.

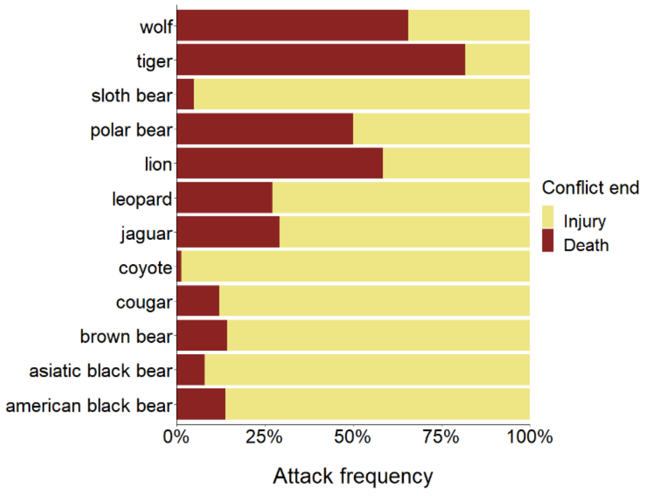

Fig D. Mortality rates per species. The data underlying this Figure can be found in S2 Data.

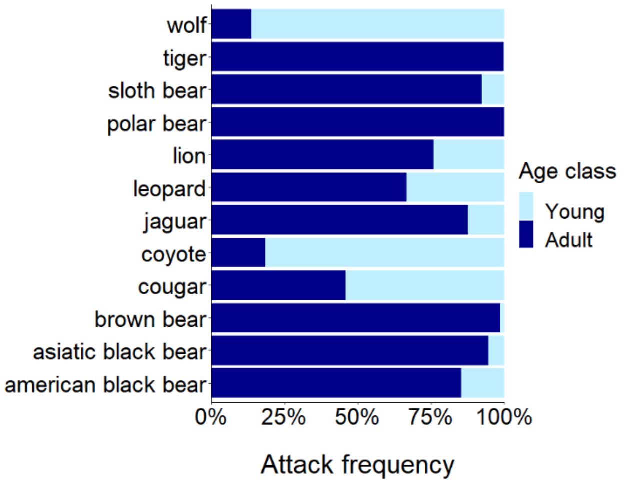

Fig E. Age class per species. The data underlying this Figure can be found in S2 Data.

Canidae 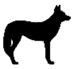

Most attacks were predatory or unprovoked (88%, n = 399; Fig A) and mainly involved children playing. Attacks ended with the death of the person in 49% of the cases (n = 270) and mainly involved children (86%, n = 419; Fig B and E).

Coyote (Canis latrans). Half of the attacks recorded were predatory/investigative (54%, n = 22), whereas in 32% of the cases one or more dogs were involved (n = 13; Fig C). Even if most attacks involved children (81%, n = 66), only 1% of the cases were fatal (n = 2; Fig D and E). In 30% of the attacks, people were carrying out various activities at home or just outside or in the yard when the attack occurred. Twenty-one percent of the people involved were walking one or more dogs, 17% of the people were walking without dogs and another 12% were engaged in sport activities.

Wolf (Canis lupus). Most of the attacks collected were predatory/unprovoked (92%, n = 377 of 410 attacks where the scenario was known), and 5% were related to the presence of food (n = 22). One percent of the attacks were provoked by the person (who was feeding, chasing, or somehow disturbing the animal), and another 1% were due to an involuntarily sudden encounter (Fig C). Of 280 cases where information on human activity was known, 75% were children playing, 12% were engaged in livelihood activities and 11% in recreational activities. Attacks almost always involved children (86%, n = 353; Fig E). In almost all predatory/unprovoked attacks that occurred in Europe and North America (15 out of 18), the animal was food-conditioned, whereas in one case it was in poor health. In more than one case where the wolf was known to be food-conditioned, the necropsy also revealed poor health conditions. Of the remaining two cases, which occurred in Alaska, one involved a pack of wolves in good health, whereas in the other case the wolf was not known and was not shot after the attack, and therefore no information was available. With respect to the cases in Israel that mainly involved children in campsites (at least 10 cases reported), it is possible that wolves had been fed by tourists; however, this information was not confirmed, as also reported by Linnell et al.[16]. It is also important to note that cases collected in India, which were made available for specific areas and years, likely represent a subsample of the actual cases and thus must be taken as a minimum number for this country. In Europe and North America, very few cases occurred (n = 25) between 1980 and 2019. In almost all predatory/unprovoked attacks (15 out of the 18 predatory/unprovoked attacks reported), the animal was known to be food-conditioned, whereas in one case necropsy revealed poor health conditions. In the other three cases where the circumstances are known, the wolf was provoked. This highlights the fact that in these regions wolves generally do not pose a threat to people unless they are disturbed, or their behaviour has been altered as a result of food conditioning or health problems, which sometimes go together. On the contrary, situations like that

found in some regions of India, where many predatory attacks involved children, provide a completely different perspective of wolf-human interactions. The reasons for such differences lie in the different socio-economic and ecological contexts[16–18].

Attack circumstances vary across regions of the world.

Human activities and scenario. We found that people were attacked under considerably different circumstances mainly depending on the region (see Fig 3, Fig 4 and main text).

Mortality rates and age of the victims. In general, injury was the most common result of conflicts in both low- and high-income countries. However, most fatal attacks (85%) occurred in lower-income countries (low- and middle low-income categories; Fig F). This is likely due to the fact that tigers and lions, whose attacks were mainly predatory and caused particularly high numbers of deaths (Fig C and D), are almost exclusively present in these regions. In addition, the fact that victim rescue and hospitalization procedures are likely slower than in higher-income regions, and sometimes completely lacking, might contribute to reducing the survival chances of the attacked person.

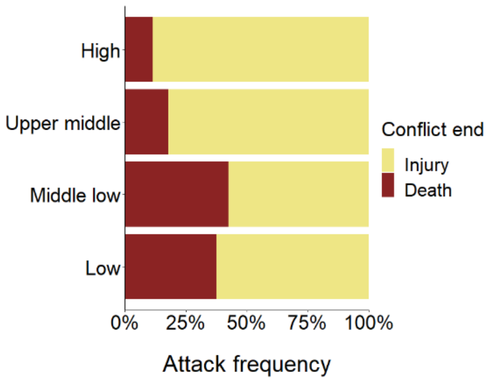

Fig F. Conflict end by income class. The data underlying this Figure can be found in S2 Data.

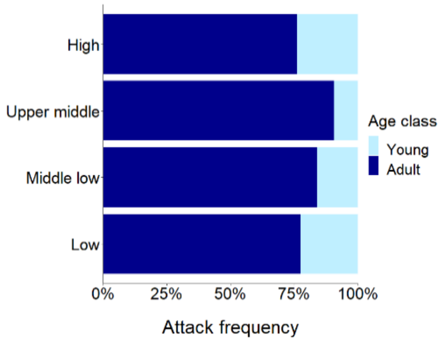

Fig G. Age class of the person/people attacked by income class. The data underlying this Figure can be found in S2 Data.

Surprisingly and contrary to our expectations, we found that children were involved in negative interactions with large carnivores almost equally in high- and low-income countries (Fig G). In particular, children were the main targets of predatory attacks by cougars and coyotes in North America and of wolves in some areas of India (Fig C in S2 File).

REFERENCES

1. Kshettry A, Vaidyanathan S, Athreya V. Leopard in a tea-cup : A study of leopard habitat-use and human-leopard interactions in north-eastern India. PLoS One. 2017;12: e0177013. doi:10.5061/dryad.pc539.Funding

2. Treves A, Naughton-Treves L. Risk and opportunity for humans coexisting with large carnivores. J Hum Evol. 1999;36: 275–282.

3. Yamazaki K, Bwalya T. Fatal lion attacks on local people in the Luangwa Valley, eastern Zambia. South African J Wildl Res. 1999;29: 19–21.

4. Packer C, Ikanda D, Kissui BM, Kushnir H. Lion attacks on humans in Tanzania. Nature. 2005;436: 791. doi:10.1038/436791a

5. Ikanda D, Kushnir H, Weisberg S, Olson E, Juntunen T, Packer C. Using landscape characteristics to predict risk of lion attacks on humans in south-eastern Tanzania Using landscape characteristics to predict risk of lion attacks on humans in south-eastern Tanzania. 2016; 524–532. doi:10.1111/aje.12157

6. Kushnir H, Leitner H, Ikanda D, Packer C. Human and Ecological Risk Factors for Unprovoked Lion Attacks on Humans in Southeastern Tanzania. Hum Dimens Wildl. 2010;15: 315–331. doi:10.1080/10871200903510999

7. Tilson R, Nyhus PJ. Tigers of the World: The Science, Politics and Conservation of Panthera tigris. 2nd editio. Press A, editor. Elsevier Science; 2009.

8. Goodrich JM. Human-tiger conflict: A review and call for comprehensive plans. Integr Zool. 2010;5: 300–312. doi:10.1111/j.1749-4877.2010.00218.x

9. Dhanwatey HS, Crawford JC, Abade LAS, Dhanwatey PH, Nielsen CK, Sillero-Zubiri C. Large carnivore attacks on humans in central India: A case study from the Tadoba-Andhari Tiger Reserve. Oryx. 2013;47: 221–227. doi:10.1017/S0030605311001803

10. Singh SK, Vipin, Mishra S, Pandey P, Kumar VP, Goyal SP. Understanding human–tiger conflict around corbett tiger reserve India: A case study using forensic genetics. Wildl Biol Pract. 2015;11: 1–11. doi:10.2461/wbp.2015.11.1

11. Gurung B, Smith JLD, McDougal C, Karki JB, Barlow A. Factors associated with human-killing tigers in Chitwan National Park, Nepal. Biol Conserv. Elsevier Ltd; 2008;141: 3069–3078. doi:10.1016/j.biocon.2008.09.013

12. DeSantis LRG, Patterson BD. Dietary behaviour of man-eating lions as revealed by dental microwear textures. Sci Rep. Springer US; 2017;7: 1–7. doi:10.1038/s41598-017-00948-5

13. Bombieri G, Naves J, Penteriani V, Selva N, Fernández-Gil A, López-Bao JV, et al. Brown bear attacks on humans: a worldwide perspective. Sci Rep. 2019;9. doi:10.1038/s41598-019-44341-w

14. Smith TS, Herrero S. Human-bear conflict in Alaska: 1880-2015. Wildl Soc Bull. 2018; 1–10. doi:10.1002/wsb.870

15. Støen OG, Ordiz A, Sahlén V, Arnemo JM, Saebo S, Mattsing G, et al. Brown bear (Ursus arctos) attacks resulting in human casualties in Scandinavia 1977–2016; management implications and recommendations. PLoS One. 2018;13. doi:10.1371/journal.pone.0196876

16. Linnell JDC, Kovtun E, Rouart I. Wolf attacks on humans: an update for 2002-2020. NINA Rep 1944 Nor Inst Nat Res. 2021;

17. Linnell JDC, Andersen R, Andersone Z, Balciauskas L, Blanco JC, Boitani L, et al. The fear of wolves: A review of wolf attacks on humans. NINA - Oppdragsmeld. 2002;731: 1–65.

18. Jhala Y V. Status, ecology and conservation of the indian wolf. J Bombay Nat Hist Soc. 2003; 2–3.
