# Supplementary material for: A worldwide perspective on large carnivore attacks on humans
Source: PLoS Biol. 2023 Jan 31;21(1):e3001946. doi: 10.1371/journal.pbio.3001946 (PMC9888692; doi:10.1371/journal.pbio.3001946)
Supplement: S2 File — Characteristics of large carnivore predatory attacks on humans documented in our study. (PDF) [file pbio.3001946.s002.pdf]

S2 File. Characteristics of large carnivore predatory attacks on humans documented in our study.

Predatory attacks represent the most dangerous type of large carnivore attack, since the predator's intention is to kill for food. Of the total cases collected globally for which the scenario was known (n = 3480), 1696 cases were classified as predatory or unprovoked, which represents 49% of all cases. We decided to group predatory and unprovoked scenarios as we did not always have enough details to consider it as predatory, but it was certainly deliberate. In any case, attacks classified as unprovoked were few (n = 9). We included investigative attacks in this category as well. This type of scenario has been described especially for canids [1,2], when the animal intentionally attacks a person, with the presumed purpose of testing or investigating them as potential prey. In such cases, often the person involved is resting or sleeping. Usually, adults are involved in these attacks and the animal does not press the attack, but readily flees after the person reacts [1]. Unprovoked (but not predatory) attacks mostly occurred in North America and Europe, where the animals involved were highly food-conditioned, which can be considered the real cause of the attack.

Because almost half of the cases collected were predatory or unprovoked, it is worth investigating such cases in detail, to identify the main characteristics and potentially important drivers that might increase the risk of such events. As mentioned in S1 File, of the three large carnivore families under study, felids and canids were the most involved in predatory cases, with 93% (n = 1227) felid attacks and 88% (n = 399) canid attacks recorded being predatory or unprovoked. Instead, bears were rarely involved in this type of encounter (4%, n = 70).

Spatio-temporal patterns

Predatory attacks recorded mainly took place in India (72% of the 1696 cases), followed by Africa (14%; Fig 4). We found high inter-species variation in seasonal trends (Fig A). For most species, months with higher attack frequencies probably represent times when carnivore and human activities overlap the most. For example, predatory attacks by American *Ursus americanus* and Asiatic *Ursus thibetanus* black bears were mainly concentrated between May and August, when both bears and people are most active outside. Similar trends were found for coyotes *Canis latrans*, wolves *Canis lupus*, cougars *Puma concolor* and jaguars *Panthera onca*, although cougar attacks were mainly concentrated in late summer months (June-September). Most lion *Panthera leo* predations were concentrated between March and May, whereas tiger *Panthera tigris* attacks occurred almost equally throughout the year, with the highest peak in April. A completely different trend was found for leopards *Panthera pardus*, whose attacks were concentrated between November and March.

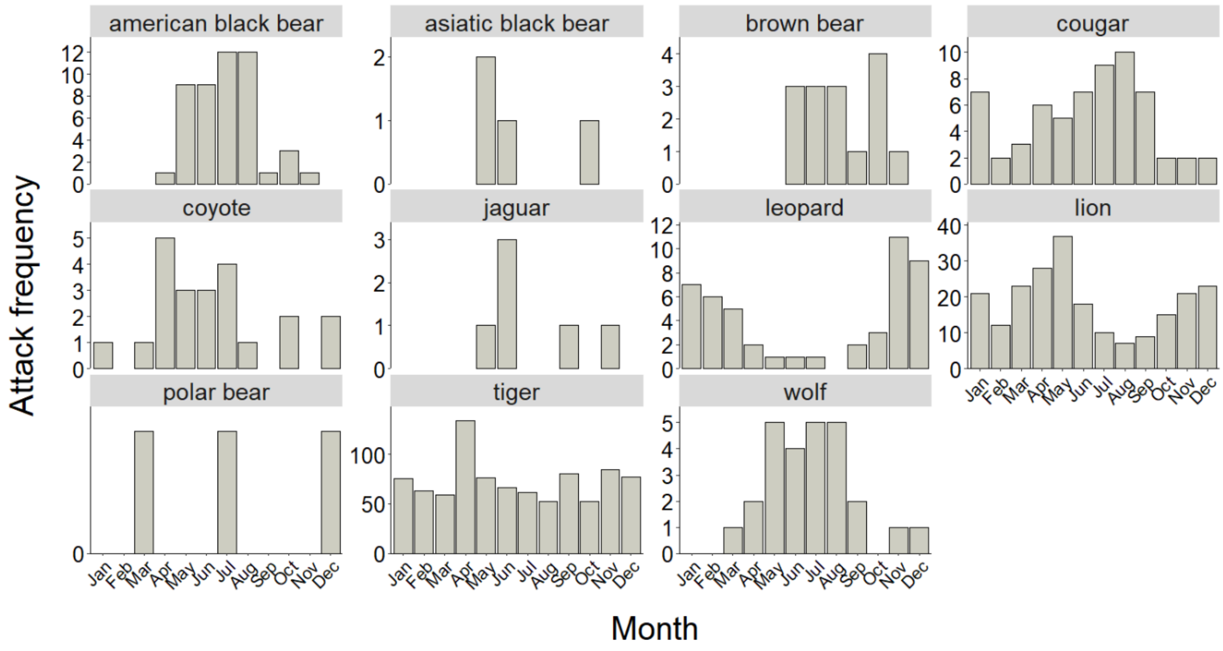

Fig A. Seasonal trend of predatory attacks by species. The data underlying this Figure can be found in S2 Data.

However, when analysing the total number of attacks as a Negative Binomial distributed model for all species over the months, we did not find any statistical difference, being the null model the best one:

Null model <- glm.nb(n~1, data=dbTimeComplete)

Season model <- glm.nb(n~month, data=dbTimeComplete)

AIC(Null model,Season model)

#	df	AIC
**#Null model**	**2**	**578.9031**
#Season model	13	596.1519

As for circadian trends of predatory attacks, we found that the best model (fitted as a Negative Binomial distributed regression model) included the interaction between species and daytime:

```
regNB1 <- glm.nb(n~daytime + species,data=dbTimeComplete)
regNB2 <- glm.nb(n~daytime,data=dbTimeComplete)
regNB3 <- glm.nb(n~species,data=dbTimeComplete)
regNB4 <- glm.nb(n~species*daytime,data=dbTimeComplete)
AIC(regNB1,regNB2,regNB3,regNB4)
```

	#df	AIC
#regNB1	13	175.0493
#regNB2	4	201.2022
#regNB3	11	183.7884
**#regNB4**	**25**	**50.0000**

The data underlying statistical models can be found in S2 Data.

Most species attacked during daytime. Only lion attacks were more frequent at night (Fig B). Information on temporal patterns of predatory attacks by wolves in India was not available.

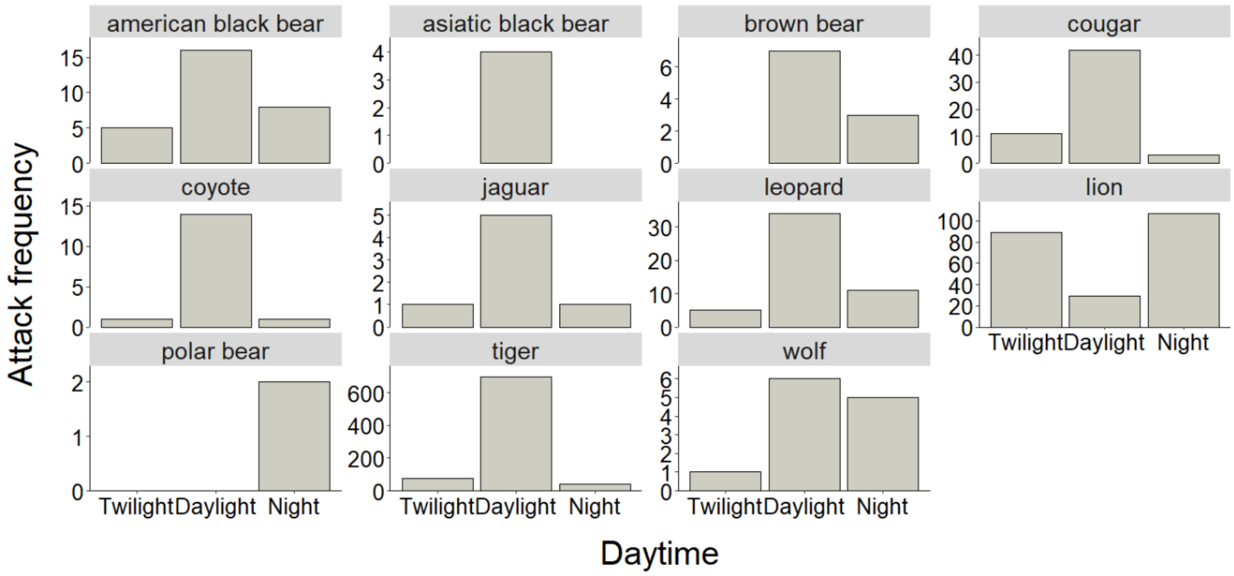

Fig B. Time of day of predatory attacks. The data underlying this Figure can be found in S2 Data.

Mortality rates and main attack characteristics

As expected, predatory attacks caused considerably more deaths compared to all other scenarios (91% of all deaths recorded). Contrary to our predictions and in contrast to that found in previous studies focusing on predatory attacks on humans in North America [3,4], victims of predatory attacks were mostly adults (70%), if considered all together. Like other attack circumstances, the age of the victim varied depending on the species and the local context. On one hand, victims of cougars, coyotes, wolves and leopards were mainly children (Fig C), which is in line with the above-mentioned previous literature. On the other hand, adults were the main targets of predation by the large felids of Asia and Africa (Fig C). One possible explanation could be that in lower-income countries, especially in those areas where felids have specialized in preying on humans, adults are more likely to be found outdoors in large carnivore habitats while carrying out livelihood activities, and thus represent easy prey, whereas children are probably not entering large carnivore habitats but remain more often in proximity of their houses and villages. This is also supported by the prevalence found in human activities during predatory attacks, which was mainly represented by people working and carryig out livelihood activities (65%). It is also important to note that, even within the same species, age prevalence varies depending on the study area.

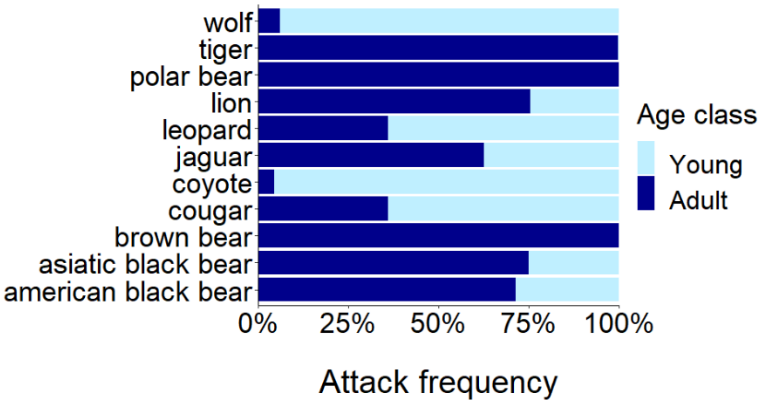

Fig C. Age class of the victims of predatory attacks by species. The data underlying this Figure can be found in S2 Data.

Similarly, we found that predatory attacks involved almost equally adults alone (46%) and in groups (44%), whereas children alone were slightly less often attacked (3%) than children in groups (7%). This is probably due to the fact that children are usually accompanied by adults when outside. Composition of the human group attacked by species is provided in Fig D. Information on the age and group composition for

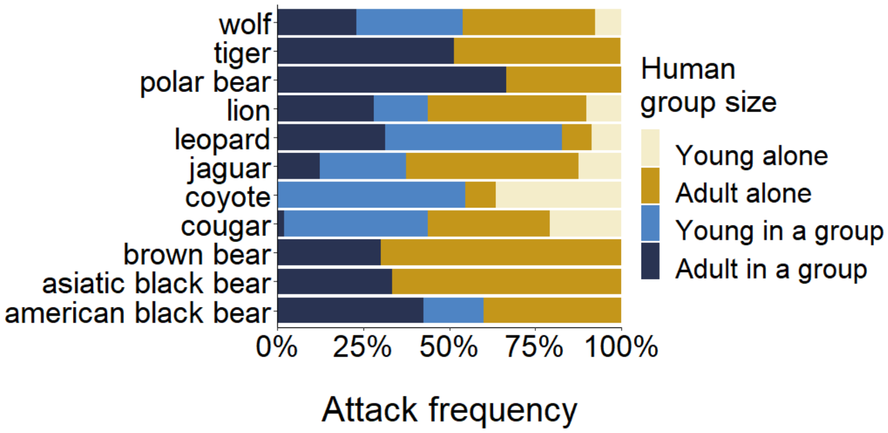

Fig D. Composition of the human group attacked by species. The data underlying this Figure can be found in S2 Data.

predatory attacks by brown bears *Ursus arctos* and wolves in India was not available.

REFERENCES

1. Linnell JDC, Andersen R, Andersone Z, Balciauskas L, Blanco JC, Boitani L, et al. The fear of wolves: A review of wolf attacks on humans. NINA - Oppdragsmeld. 2002;731: 1–65.

2. Behdarvand N, Kaboli M. Characteristics of Gray Wolf Attacks on Humans in an Altered Landscape in the West of Iran. Hum Dimens Wildl. 2015;20: 112–122. doi:10.1080/10871209.2015.963747

3. Penteriani V, Bombieri G, Fedriani JM, López-Bao JV, Garrote PJ, Russo LF, et al. Humans as prey : coping with large carnivore attacks using a predator – prey interaction perspective. Human–Wildlife Interact. 2017;11: 192–207.

4. Bombieri G, Delgado M del M, Russo LF, Garrote PJ, López-Bao JV, Fedriani JM, et al. Patterns of wild carnivore attacks on humans in urban areas. Sci Rep. 2018;8: 1–9. doi:10.1038/s41598-018-36034-7
