# Supplementary material for: A worldwide perspective on large carnivore attacks on humans
Source: PLoS Biol. 2023 Jan 31;21(1):e3001946. doi: 10.1371/journal.pbio.3001946 (PMC9888692; doi:10.1371/journal.pbio.3001946)
Supplement: S1 Table — Details of large carnivore attacks on humans collected between 1950 and 2019 on a worldwide scale. (PDF) [file pbio.3001946.s003.pdf]

Supplementary Table 1. Details of large carnivore attacks on humans collected between 1950 and 2019 on a worldwide scale.

Family	Species	Country/ Region	Income class [1]	Number of attacks recorded	Fatality rate [2]	Main scenarios [3]
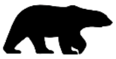 *Ursidae* N = 3192	American black bear *Ursus americanus* (1958-2017) N = 403	U.S.	H	242	0.07	Dog-related (36%)<br>Female with cubs (21%)<br>Food-related (20%)<br>(n$_{\text{known scenario}}$ = 136)
		Canada	H	160	0.24	Predatory (53%)<br>Dog-related (37%)<br>(n$_{\text{known scenario}}$ = 49)
	Brown bear [4] *Ursus arctos* (2000-2015) N = 664	U.S.	H	113	0.12	Female with cubs (67%)<br>Dog-related (13%)<br>(n$_{\text{known scenario}}$ = 61)
		Canada	H	70	0.14	Female with cubs (74%)<br>(n$_{\text{known scenario}}$ = 23)
		Europe	H [5]	291	0.07	Female with cubs (32%)<br>Involuntary sudden encounter (21%)<br>Dog-related (15%)<br>(n$_{\text{known scenario}}$ = 209)
		Iran	UM	25	0	Involuntary sudden encounter (22%)<br>Female with cubs (12%)<br>(n$_{\text{known scenario}}$ = 25)
		Turkey	UM	54	0.2	Involuntary sudden encounter (44%)<br>Food-related (40%)<br>(n$_{\text{known scenario}}$ = 52)
		Russian Federation	UM	111	0.37	Female with cubs (17%)<br>Wounded animal (16%)<br>Predatory (15%)<br>Involuntary sudden encounter (12%)<br>(n$_{\text{known scenario}}$ = 58)
	Asiatic black bear *Ursus thibetanus* N = 765	Bhutan (2006-2018)	LM	5	0.2	Unknown
		Nepal (1998-2017)	L	21	0.48	Involuntary sudden encounter (90%)<br>Animal voluntarily disturbed (10%)<br>(n$_{\text{known scenario}}$ = 21)
		India (1987-2018)	LM	610	0.06	Involuntary sudden encounter (59%)<br>Food-related (36%)<br>(n$_{\text{known scenario}}$ = 360)
		Pakistan (2014-2017)	LM	9	0.22	Female with cubs (50%)<br>Predatory (50%)<br>(n$_{\text{known scenario}}$ = 2)
		Iran (2012-2019)	UM	14	0.07	Food-related (60%)<br>Involuntary sudden encounter (27%)<br>Female with cubs (13%)<br>(n$_{\text{known scenario}}$ = 14)
		Japan (1985-2018)	H	71	0.2	Involuntary sudden encounter (43%)<br>Female with cubs (29%)<br>Predatory (21%)<br>(n$_{\text{known scenario}}$ = 14)
		Russian Federation (1983-2019)	UM	35	0.09	Involuntary sudden encounter (50%)<br>Wounded animal (21%)<br>Female with cubs (14%)<br>(n$_{\text{known scenario}}$ = 28)
	Sloth bear *Melursus ursinus*	India (1968-2017)	LM	918	0.07	Involuntary sudden encounter (61%)<br>Female with cubs (18%)<br>Wounded animal (14%)<br>(n$_{\text{known scenario}}$ = 593)
		Nepal	L	147	0.03	Unknown

	N = 1337	(1999-2015)				
		Sri Lanka<br>(1938-2004)	LM	272	0.01	Involuntary sudden encounter (65%)<br>Female with cubs (26%)<br>($n_{\text{known scenario}}$ = 62)
	**Polar bear**<br>*Ursus maritimus*<br>N = 23	Alaska<br>(1990)	H	1	1	Predatory (100%)<br>($n_{\text{known scenario}}$ = 1)
		Canada<br>(1999-2018)	H	11	0.27	Predatory (100%)<br>($n_{\text{known scenario}}$ = 1)
		Svalbard<br>(1994-2018)	H	7	0.57	Predatory (100%)<br>($n_{\text{known scenario}}$ = 1)
		Russian Federation<br>(2011-2016)	UM	4	0.5	Food-related (100%)<br>($n_{\text{known scenario}}$ = 1)
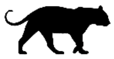<br>*Felidae*<br>N = 1694	**Cougar**<br>*Puma concolor*<br>N = 135	Canada<br>(1980-2015)	H	51	0.09	Predatory (100%)<br>($n_{\text{known scenario}}$ = 19)
		U.S.<br>(1980-2016)	H	76	0.11	Predatory (91%)<br>($n_{\text{known scenario}}$ = 34)
		Brazil<br>(2011-2016)	UM	3	0.33	Predatory (100%)<br>($n_{\text{known scenario}}$ = 3)
		Chile<br>(1989-2015)	UM	2	0.5	Predatory (100%)<br>($n_{\text{known scenario}}$ = 2)
		Mexico<br>(2014-2015)	UM	3	0	Predatory (33%)<br>Involuntary sudden encounter (33%)<br>Food-related (33%)<br>($n_{\text{known scenario}}$ = 3)
	**Jaguar**<br>*Panthera onca*<br>N = 25	Brazil<br>(2007-2018)	UM	11	0.36	Predatory (60%)<br>Food-related (20%)<br>Dog-related (10%)<br>Involuntary sudden encounter (10%)<br>($n_{\text{known scenario}}$ = 10)
		Guyana<br>(2014)	LM	1	1	Predatory (100%)<br>($n_{\text{known scenario}}$ = 1)
		Venezuela<br>(1985-2015)	UM	13	0.17	Animal voluntarily disturbed (83%)<br>Predatory (17%)<br>($n_{\text{known scenario}}$ = 6)
	**Leopard**<br>*Panthera pardus*<br>N = 205	Botswana<br>(2018)	UM	1	0	Predatory (100%)<br>($n_{\text{known scenario}}$ = 1)
		Kenya<br>(1996-2017)	L	24	0.08	Predatory<br>Involuntary sudden encounter<br>Animal voluntarily disturbed<br>(Frequencies are not known)
		Iran<br>(2008-2018)	UM	27	0.07	Involuntary sudden encounter (85%)<br>Predatory (11%)<br>($n_{\text{known scenario}}$ = 27)
		India<br>(1996-2017)	LM	117	0.54	Predatory (88%)<br>Involuntary sudden encounter (10%)<br>($n_{\text{known scenario}}$ = 52)
		Nepal<br>(2000-2010)	L	36	0	Unknown
	**African lion**<br>*Panthera leo*<br>N = 282	Kenya<br>(1990-2017)	L	51	0.18	Unknown
		Tanzania<br>(1990-2006)	L	225	0.67	Predatory (100%)<br>($n_{\text{known scenario}}$ = 225)
		Zimbabwe<br>(1999-2017)	L	6	1	Predatory (100%)<br>($n_{\text{known scenario}}$ = 3)
	**Tiger**<br>*Panthera tigris*<br>N = 1047	India (Sundarbans)<br>(1985-2015)	LM	869	0.89	Predatory (100%)<br>($n_{\text{known scenario}}$ = 869)
		Nepal<br>(1999-2016)	L	119	0.53	Unknown
		Russian Federation<br>(1980-2019)	UM	59	0.32	Wounded animal (48%)<br>Involuntary sudden encounter (20%)<br>Predatory (19%)<br>($n_{\text{known scenario}}$ = 54)

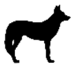 *Canidae* N = 554	**Coyote** *Canis latrans* N = 140	Canada (2001-2016)	H	11	0.09	Predatory/unprovoked (100%) ($n_{known\ scenario}$ = 3)
		U.S. (1980-2018)	H	129	0	Predatory/unprovoked (49%) Dog-related (33%) ($n_{known\ scenario}$ = 38)
	**Wolf** [6] *Canis lupus* N = 414	Iran [7] (2001-2017)	UM	59	0.10	Predatory (80%) Food-related (17%) ($n_{known\ scenario}$ = 59)
		Turkey (1993-2018)	UM	18	0.06	Food-related (67%) Involuntary sudden encounter (28%) ($n_{known\ scenario}$ = 18)
		India [8] (various periods)	LM	>302	0.87 [9]	Predatory (100%) ($n_{known\ scenario}$ = 302)
		Europe [10] (1980-2019)	H	8	0	Predatory/unprovoked (67%) Animal voluntarily provoked (33%) ($n_{known\ scenario}$ = 6)
		U.S. (1980-2019)	H	4	0.25	Predatory/unprovoked (100%) ($n_{known\ scenario}$ = 4)
		Canada (1980-2019)	H	13	0.08	Predatory/unprovoked (91%) ($n_{known\ scenario}$ = 11)
		Israel (2017)	H	10	0	Predatory/unprovoked (100%) ($n_{known\ scenario}$ = 10)

[1] L = low income (≤ $1,005); LM = lower middle income ($1,006 - 3,975); UM = upper middle income ($3,976 - 12,275); H = high income (> $12,275). Classification based on gross national income (GNI) per capita in US dollars in a year made by the World Bank Group [1].

[3] For simplicity, only scenarios more frequent than 10% are presented in the table. Percentages are calculated for the total number of cases for which the scenario is known (named n known scenario).

[4] Information was extracted from Bombieri et al. (2019) [2].

[5] For simplicity, we classified Europe as H in this table because, among European countries where attacks were collected, only Latvia and North Macedonia are not classified as H, as they are classified as UM.

[6] Numbers of wolf attacks are accurate for Europe and North America, where few cases occurred. Numbers represent a good subsample for Middle Eastern countries, whereas they represent only a small subsample for India, where many cases are known to have occurred, but detailed information was not available. For the Russian Federation, we adopted a conservative approach and decided not to report any cases, since it is almost impossible to distinguish between rabid and non-rabid cases.

[7] Information was extracted from Behdarvand and Kaboli (2015)[3] and other sources.

[8] Cases for this area are largely incomplete and referred to specific periods and areas, thus the numbers must be interpreted as minimums only. Information was extracted from Rajpurohit (1999)[4], Jhala and Sharma (1997)[5], and Jhala (2000)[6].

[9] Mortality rate was calculated for a total of 297 cases where attack end was known. Victims were children.

[10] Four attacks in Poland in 2018, where one wolf was involved in 3 of the cases. In all cases, wolves were food-conditioned and were not afraid of people. Two cases in Latvia (1998 and 2000) with no

information on the scenario. One case in Spain (1983), where the wolf was voluntarily provoked. One case in North Macedonia (2016), where a man was attacked after trying to defend his barn from a wolf. No confirmed attacks (non-rabid) occurred in the following European countries between 1980-2019: Norway, Sweden, Finland, Slovenia, Croatia, Greece, Romania, Ukraine, Estonia, Belarus, Slovakia, Bulgaria, France, Germany, Austria, Switzerland, Lithuania, Portugal, and Italy.

References

1. World Bank Group. [cited 30 Apr 2020]. Available: https://www.worldbank.org/
2. Bombieri G, Naves J, Penteriani V, Selva N, Fernández-Gil A, López-Bao J V., et al. Brown bear attacks on humans: a worldwide perspective. Sci Rep. 2019;9: 8573. doi:10.1038/s41598-019-44341-w
3. Behdarvand N, Kaboli M. Characteristics of Gray Wolf Attacks on Humans in an Altered Landscape in the West of Iran. Hum Dimens Wildl. 2015;20: 112–122. doi:10.1080/10871209.2015.963747
4. Rajpurohit KS. Child Lifting: Wolves in Hazaribagh, India. Ambio. 1999;28: 162–166.
5. Jhala Y V., Sharma DK. Child Lifting by Wolf in Eastern Uttar Pradesh, India. J Wildl Res. 1997; 94–101.
6. Jhala Y V. Status, ecology and conservation of the indian wolf. J Bombay Nat Hist Soc. 2003; 2–3.
