# Supplementary material for: A worldwide perspective on large carnivore attacks on humans
Source: PLoS Biol. 2023 Jan 31;21(1):e3001946. doi: 10.1371/journal.pbio.3001946 (PMC9888692; doi:10.1371/journal.pbio.3001946)
Supplement: S2 Table — Main published literature collected and analyzed regarding large carnivore attacks, organized by species. (PDF) [file pbio.3001946.s004.pdf]

Supplementary Table 2. Main published literature collected and analysed regarding large carnivore attacks, organized by species.

Species family	Species	Bibliography
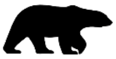<br>*Ursidae*	American black bear<br>*Ursus americanus*	Herrero, S., Higgins, A. (2003). Human injuries inflicted by bears in Alberta: 1960–98. *Ursus* 14: 44–54.
		Herrero, S., Higgins, A., Cardoza, J. E. et al. (2011). Fatal attacks by American black bear on people: 1900–2009. Journal of Wildlife Management 75: 596–603.
	Brown bear<br>*Ursus arctos*	Ambarli U., Bilgin C. (2008). Human–brown bear conflicts in Artvin, northeastern Turkey: Encounters, damage, and attitudes. *Ursus* 19(2):146–153.
		Bombieri, G., Naves, J., Penteriani, V., et al. (2019). Brown bear attacks on humans: a worldwide perspective. Scientific Reports 9: 8573.
		Kudrenko, S., Ordiz, A., Barysheva S. et al. (2020). Human injuries and fatalities caused by brown bears in Russia, 1932–2017. *Wildlife Biology* 1.
		Herrero, S., Higgins, A. (2003). Human injuries inflicted by bears in Alberta: 1960–98. *Ursus* 14: 44–54.
		Støen, O. G., Ordiz, A., Sahlén, V., et al. (2018). Brown bear (Ursus arctos) attacks resulting in human casualties in Scandinavia 1977–2016; management implications and recommendations. PLoS ONE 13: e0196876.
	Asiatic black bear<br>*Ursus thibetanus*	Akiyama, G., Kuwahara, H., Asahi, R. et al. (2017). Prompt procedures have a great impact on the consequences of Asiatic black bear mauling. Journal of Nippon Medical School **84**: 294–300.
		Ali, A., Waseem, M., Teng, M. et al. (2018). Human–Asiatic black bear (Ursus thibetanus) interactions in the Kaghan Valley, Pakistan. Ethology Ecology and Evolution 30: 399–415.
		Charoo, S. A., Sharma, L. K., Sathyakumar, S. (2011). Asiatic black bear–human interactions around Dachigam National Park, Kashmir, India. Ursus 22: 106–113.
		Chauhan N.P.S. (2003). Human casualties and livestock depredation by black and brown bears in the Indian Himalaya, 1989-98. *Ursus* 14(1): 84-8.
		Choudhury S., Ali M., Mubashir T. et al. (2008). Predator Alert - Attacks on humans by leopards and Asiatic black bear in the Kashmir valley - Analysis of case studies and spatial patterns of elevated conflict. Wildlife Trust of India.
		Jamtsho, Y., Wangchuk, S. (2016). Assessing patterns of human–Asiatic black bear interaction in and around Wangchuck Centennial National Park, Bhutan. Global Ecology and Conservation 8: 183–189.
		Japan Bear Network. (2011). Report on statistics of the bear caused human injuries (in Japanese). Ibaraki: Japan Bear Network.
		Lal Moten T., Bhat T. A., Gulzar A. et al. (2017). Causalities of human wildlife conflict in Kashmir valley, India; a neglected form of trauma: our 10 year study. *International Journal of Research in Medical Sciences* 55: 1898–1902.
		Nabi D.G., Tak S.R., Kangoo K.A., Halwai M.A. (2009). Comparison of injury pattern in victims of bear (Ursus thibetanus) and leopard (Panthera pardus) attacks. A study from a tertiary care center in Kashmir. *European Journal of Trauma and Emergency Surgery* 35:153-158.
		Oshima, T., Ohtani, M., Mimasaka, S. (2018). Injury patterns of fatal bear attacks in Japan: a

		description of seven cases. Forensic Science International 286: e14–e19.<br><br>Rasool, A., Wani, A. H., Darzi, M. A., et al. (2010). Incidence and pattern of bear maul injuries in Kashmir. Injury 41: 116–119.<br><br>Shah, A., Mir, B., Ahmad, I., et al. (2010). Pattern of bear maul maxillofacial injuries in Kashmir. National Journal of Maxillofacial Surgery 1: 96.<br><br>Tak, S. R., Nabi, D. G., Halwai, M. A. & Mir, B. A. (2009). Injuries from bear (Ursus thibetanus) attacks in Kashmir. Turkish Journal of Trauma & Emergency Surgery 15: 130–134.<br><br>Yamazaki, K. (2004). Recent bear–human conflicts in Japan. International Bear News 13:16-17.<br><br>Yamazaki, K. (2017). Consecutive fatal attacks by Asiatic black bear on humans in Northern Japan. International Bear News 26: 16–17.
	Sloth bear<br><br>*Melursus ursinus*	Akhtar, N. (2006). Human–sloth bear conflict: a threat to sloth bear conservation. International Bear News 15:15–17.<br><br>Bargali, H. S., Akhtar, N. & Chauhan, N. P. S. (2005). Characteristics of sloth bear attacks and human casualties in North Bilaspur Forest Division, Chhattisgarh, India. Ursus 16: 263–267.<br><br>Debata, S., Swain, K. K., Sahu, H. K., Palei, H. S. (2017). Human–sloth bear conflict in a human-dominated landscape of northern Odisha, India. Ursus 27:90–98.<br><br>Dhamorikar, A. H., Mehta, P., Bargali, H., Gore, K. (2017). Characteristics of human–sloth bear (Melursus ursinus) encounters and the resulting human casualties in the Kanha–Pench corridor, Madhya Pradesh, India. PLoS ONE 12: 1–18.<br><br>Garcia, K. C., Joshi, H. M., Dharaiya, N. (2016). Assessment of human–sloth bear conflicts in North Gujarat, India. Ursus 27: 5–10.<br><br>Jangid, K. A., Sharma, K. R. (2018). How locals characterize the causes of sloth bear attacks in Jawai, Rajasthan. International Bear News 27: 11–12.<br><br>Mardaraj, P., Dutta, S.K. (2011). Human–sloth bear conflict in Balasore Forest Division, Eastern India. Saarbrücken, Germany: LAP Lambert Academic Publishing GmbH & Co. KG.<br><br>Mardaraj, P. C. (2015). Identifying key issues for the conservation of sloth bear (Melursus ursinus) in Rajnilgiri, Odisha, Eastern India. The Rufford Foundation, United Kingdom.<br><br>Rajpurohit, K. S., Krausman, P. R. (2000). Human–sloth bear conflicts in Madhya Pradesh, India. Wildlife Society Bulletin 28: 393–399.<br><br>Ratnayeke, S., Van Manen, F. T., Pieris, R. & Pragash, V. S. J. (2014). Challenges of large carnivore conservation: sloth bear attacks in Sri Lanka. Human Ecology 42:467–479.<br><br>Sharp, T., Sonone, S. D. (2011). Sloth bear attacks: causes and consequences. International Bear Newsletter 20: 14–17.<br><br>Sharp, T. R., Swaminathan, S., Arun, A. S., et al. (2017). Sloth bear attack behavior and a behavioral approach to safety. Final report to International Association for Bear Research and Management.<br><br>Singh N., Sonone S., Dharaiya N. (2018). Sloth bear attacks on humans in central India: implications for species conservation. Human–Wildlife Interactions 12(3):338–347.
	Polar bear<br><br>*Ursus maritimus*	Clark, D. (2003). Polar bear–human interactions in Canadian national parks, 1986–2000. Ursus 14: 65–71.<br><br>Wilder, J. M., Vongraven, D., Atwood, T., et al. (2017). Polar bear attacks on humans: implications of a changing climate. Wildlife Society Bulletin 41:537–547.
	Cougar	Beier, P. 1991. Cougar attacks on humans in the United States and Canada. *Wildlife Society Bulletin* 19:403–412.

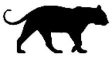<br>*Felidae*	*Puma concolor*	Coss R.G., Fitzhugh E.L., Schmid-Holmes S. et al. (2009). The Effects of Human Age, Group Composition, and Behavior on the Likelihood of Being Injured by Attacking Pumas. *Anthrozoös* 22:1, 77-87.<br><br>Mattson D., Logan K., Sweanor L. (2011). Factors governing risk of cougar attacks on humans. *Hum Wildl Interact J* 5:135–158.
	Jaguar<br><br>*Panthera onca*	Hoogesteijn R., Hoogesteijn A.L., Tortato F. et al. (2016). Consideraciones sobre la peligrosidad del jaguar para los humanos: ¿quién es letal para quién? In: Castaño-Uribe, C., C. A. Lasso, R. Hoogesteijn, A. Diaz-Pulido y E. Payán (Editores). II. Conflictos entre felinos y humanos en América Latina. Serie Editorial Fauna Silvestre Neotropical. Instituto de Investigación de Recursos Biológicos Alexander von Humboldt (IAvH), Bogotá, D. C., Colombia.<br><br>Iserson, K.V., Francis, A.M. (2015). Jaguar Attack on a Child: Case Report and Literature Review. *Western Journal of Emergency Medicine: Integrating Emergency Care with Population Health* 16(2): 303-309.<br><br>Jędrzejewski W., Carreño R., Sánchez-Mercado A. et al. (2017). Human-jaguar conflicts and the relative importance of retaliatory killing and hunting for jaguar (Panthera onca) populations in Venezuela, *Biological Conservation* 209: 524-532.<br><br>Neto M.F.C, Neto D.G., Haddad V. (2011). Attacks by Jaguars (Panthera onca) on Humans in Central Brazil: Report of Three Cases, with Observation of a Death. *Wilderness & Environmental Medicine* 22, 130–135.
	Leopard<br><br>*Panthera pardus*	Acharya K.P., Kumar Paudel P., Jnawali S.R. et al. (2017). Can forest fragmentation and configuration work as indicators of human–wildlife conflict? Evidences from human death and injury by wildlife attacks in Nepal. *Ecological Indicators* 80: 74-83.<br><br>Athreya, V.R., Thakur, S.S., Chaudhuri, S. & A.V. Belsare. (2004). A study of the man-leopard conflict in the Junnar Forest Division, Pune District, Maharashtra. Submitted to the Office of the Chief Wildlife Warden, Maharashtra State Forest Department, and the Wildlife Protection Society of India, New Delhi, India.<br><br>Athreya V., Odden M., Linnell J.D.C, Karanth K.L (2010). Translocation as a Tool for Mitigating Conflict with Leopards in Human-Dominated Landscapes of India. *Conservation Biology* 25(1):133-41.<br><br>Athreya V., Isvaran K., Odden M. et al. (2020). The impact of leopards (Panthera pardus) on livestock losses and human injuries in a human-use landscape in Maharashtra, India. *PeerJ* 8:e8405.<br><br>Chattha S.A., Iqbal S., Rasheed Z. et al. (2013). Human-leopard conflict in Machiara National Park (MNP), Azad Jamu and Kashmir (AJ and K), Pakistan. *J. Glob. Innov. Agric. Soc. Sci.* 1(1): 17-21.<br><br>Choudhury S., Ali M., Mubashir T. et al. (2008). Predator Alert - Attacks on humans by leopards and Asiatic black bear in the Kashmir valley - Analysis of case studies and spatial patterns of elevated conflict. Wildlife Trust of India.<br><br>Dhanwatey, H., Crawford, J., Abade et al. (2013). Large carnivore attacks on humans in central India: A case study from the Tadoba-Andhari Tiger Reserve. *Oryx* 47(2), 221-227 (leopard & tiger)<br><br>Durrheim D.N.D., Leggat P.A (1999). Risk to tourists posed by wild mammals in South Africa. *Journal of Travel Medicine* 6: 172-179.<br><br>Khan U., Lovari S., Shah S.A., Ferretti F. (2018). Predator, prey and humans in a mountainous area: loss of biological diversity leads to trouble. *Biodiversity and Conservation* 27:2795–2813.

		Kshettry A., Vaidyanathan S., Athreya V.R. (2017). Leopard in a tea-cup: A study of leopard habitat-use and human-leopard interactions in north-eastern India. *PLoS ONE* 12(5): e0177013.<br><br>Kumar P., Chandel S., Kumar V., Sankhyan V. (2017). Leopard–Human Conflict Led Casualties and Conservation Awareness Campaign in Shivalik Hills of Northern India. *Proc. Natl. Acad. Sci., India, Sect. B Biol. Sci*. 87(3):893–898.<br><br>Lal Moten T., Bhat T. A., Gulzar A. et al. (2017). Causalities of human wildlife conflict in Kashmir valley, India; a neglected form of trauma: our 10 year study. *International Journal of Research in Medical Sciences* 55: 1898–1902.<br><br>Nabi, D.G., Tak S.R., Kangoo K.A., Halwai M.A (2009). Injuries from leopard attacks in Kashmir. Injury 40 (1):90-92.<br><br>Nabi D.G., Tak S.R., Kangoo K.A., Halwai M.A. (2009). Comparison of injury pattern in victims of bear (Ursus thibetanus) and leopard (Panthera pardus) attacks. A study from a tertiary care center in Kashmir. *European Journal of Trauma and Emergency Surgery* 35:153-158.
	African lion<br><br>*Panthera leo*	DeSantis L.R.G., Patterson B.D. (2017). Dietary behaviour of man-eating lions as revealed by dental microwear textures. *Scientific Reports* 7: 904.<br><br>Dunham K.M., Ghiurghi A., Cumbi R., Urbano F. (2010). Human–wildlife conflict in Mozambique: a national perspective, with emphasis on wildlife attacks on humans. *Oryx* 44(2):185–193.<br><br>Durrheim D.N.D., Leggat P.A (1999). Risk to tourists posed by wild mammals in South Africa. *Journal of Travel Medicine* 6: 172-179.<br><br>Kushnir H., Leitner H., Ikanda D., Packer C. (2010): Human and Ecological Risk Factors for Unprovoked Lion Attacks on Humans in Southeastern Tanzania. *Human Dimensions of Wildlife: An International Journal* 15(5):315-331.<br><br>Kushnir H., Weisberg S., Olson E. et al. (2014). Using landscape characteristics to predict risk of lion attacks on humans in south-eastern Tanzania. *Afr. J. Ecol.* 52: 524–532.<br><br>Matema S., Andersson J.A. (2015). Why are lions killing us? Human–wildlife conflict and social discontent in Mbire District, northern Zimbabwe. *The Journal of Modern African Studies* 53: 93-120.<br><br>Mukeka J.M., Ogutu J.O., Kanga E., Røskaft E. (2018). Characteristics of Human-Wildlife Conflicts in Kenya: Examples of Tsavo and Maasai Mara Regions. *Environment and Natural Resources Research* 8(3):148-165.<br><br>Packer, C., Ikanda, D., Kissui, B. & Kushnir, H. (2005) Lion attacks on humans in Tanzania. *Nature* 436:927–928.<br><br>Packer C., Swanson A., Ikanda D., Kushnir H. (2011). Fear of Darkness, the Full Moon and the Nocturnal Ecology of African Lions. *PLoS ONE* 6(7): e22285.<br><br>Peterhans J.C.K., Gnoske T.P. (2001). The Science of 'Man-Eating' Among Lions Panthera leo With a Reconstruction of the Natural History of the 'Man-Eaters of Tsavo'. *Journal of East African Natural History* 90(1):1-40.<br><br>Yamazaki K., Bwalya T. (1999). Fatal lion attacks on local people in the Luangwa Valley, Eastern Zambia. *S.Afr.J.Wild.Res* 29(1):19-21.<br><br>Yeakel J.D., Patterson B.D., Fox-Dobbs K. et al. (2009). Cooperation and individuality among man-eating lions. *PNAS* 106(45):19040-19043.
	Tiger	Acharya K.P., Kumar Paudel P., Jnawali S.R. et al. (2017). Can forest fragmentation and configuration work as indicators of human–wildlife conflict? Evidences from human death and

	Panthera tigris	injury by wildlife attacks in Nepal. *Ecological Indicators* 80: 74-83.<br><br>Barlow A.C.D., Ahmad I., Smith J.L.D. (2013). Profiling Tigers (Panthera tigris) to Formulate Management Responses to Human-Killing in the Bangladesh Sundarbans. *Wildl. Biol. Pract.* 9(2): 30-39.<br><br>Bhattarai B., Fischer K. (2014). Human–tiger Panthera tigris conflict and its perception in Bardia National Park, Nepal. *Oryx* 1-7.<br><br>Das C.S. (2012). Tiger straying incidents in Indian Sundarban: statistical analysis of case studies as well as depredation caused by conflict. *Eur J Wildl Res* 58:205–214.<br><br>Das C.S., Bandyopadhyay S. (2012). Sharing Space: Human-Animal Conflicts in Indian Sundarban. Progressive Publishers, Kolkata.<br><br>Das C.S. (2015). Causes, Consequences and Cost-Benefit Analysis of the Conflicts Caused by Tiger Straying Incidents in Sundarban, India. *Proceedings of the Zoological Society* 68 (2): 120-130.<br><br>Das C.S. (2017). Human Wildlife Conflicts in Sundarban, State Art Report on Biodiversity in Indian Sundarban. WWF, New Delhi.<br><br>Dhanwatey, H., Crawford, J., Abade, L. et al. (2013). Large carnivore attacks on humans in central India: A case study from the Tadoba-Andhari Tiger Reserve. *Oryx* 47(2), 221-227.<br><br>Dhungana R., Savini T., Karki J.B. et al. (2018). Living with tigers Panthera tigris: patterns, correlates, and contexts of human–tiger conflict in Chitwan National Park, Nepal. *Oryx* 52(1), 55-65.<br><br>Goodrich J.M., Miquelle D.G. (2005). Translocation of problem Amur tigers Panthera tigris altaica to alleviate tiger-human conflicts. *Oryx* 39(4): 454–457.<br><br>Goodrich J.M. (2010). Human–tiger conflict: A review and call for comprehensive plans. *Integrative Zoology* 5: 300-312.<br><br>Goodrich J.M., Seryodkin I., Miquelle D.G., Bereznuk S.L. (2011). Conflicts between Amur (Siberian) tigers and humans in the Russian Far East. *Biological Conservation* 144: 584-592.<br><br>Gupta, A.C. (1964). Wildlife of lower Bengal with particular reference to Sundarbans. In: West Bengal Forests: Centenary Commemoration Volume, Forest Directorate, Government of West Bengal: 233–238.<br><br>Gurung B., Smith J.L.D., McDougal C. et al. (2008). Factors associated with human-killing tigers in Chitwan National Park, Nepal. *Biological Conservation* 141: 3069-3078.<br><br>Lamichhane B.R., Persoon G.A., Leirs H. et al. (2017). Are conflict-causing tigers different? Another perspective for understanding human-tiger conflict in Chitwan National Park, Nepal. *Global Ecology and Conservation* 11:177-187.<br><br>Lubis M. I., Pusparini W., Prabowo S. A. et al. (2020). Unraveling the complexity of human–tiger conflicts in the Leuser Ecosystem, Sumatra. *Anim Conserv* 23: 741-749.<br><br>Neumann-Denzau G., Denzau H. (2010). Examining certain aspects of human-tiger conflict in the Sundarbans Forest, Bangladesh. *Tigerpaper* 37(3).<br><br>Nikolaev I.G. (2014). Tiger attacks on humans in Primorsky (Ussuri) Krai in XIX-XXI centuries. (In Russian).<br><br>Singh S.K., Vipin, Mishra S., Pandey P., Kumar V.P., Goyal S.P. (2015). Understanding Human–

		Tiger Conflict around Corbett Tiger Reserve India: A Case Study Using Forensic Genetics. *Wildl. Biol. Pract.* 11(1): 1-11.<br><br>Singh R., Nigam P., Qureshi Q. et al. (2015). Characterizing human–tiger conflict in and around Ranthambhore Tiger Reserve, western India. *Eur J Wildl Res* 61:255–261.<br><br>Tan C.K.W., O'Dempsey T., Macdonald D.W., Linkie M. (2015). Managing present day large-carnivores in 'island habitats': lessons in memoriam learned from human-tiger interactions in Singapore. *Biodivers Conserv* 24:3109–3124.<br><br>Tilson, R., Nyhus, P. J. Tigers of the World: The Science, Politics and Conservation of Panthera tigris. (Elsevier Science, 2009).<br><br>Wegge P., Kumar Y.S., Lamichhane B.R. (2018). Are corridors good for tigers Panthera tigris but bad for people? An assessment of the Khata corridor in lowland Nepal. *Oryx* 52(1): 35–45.
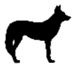<br><br>*Canidae*	Coyote<br><br>*Canis latrans*	Baker, R. O., Timm, R. M. (2017). Coyote attacks on humans. 1970–2015: implications for reducing the risks. *Human–Wildlife Interactions* 11, 120–132.<br><br>Carbyn, L. N. (1989). Coyote attacks on children in western North America. *Wildlife Society Bulletin* 172:444–446.<br><br>Lukasik, V. M., Alexander, S. M. (2011). Human-Coyote interactions in Calgary, Alberta. *Hum. Dimens. Wildl.* 16, 114–127.<br><br>Poessel, S. A., Breck, S., Teel, T. L. et al. (2013). Patterns of Human – Coyote Conflicts in the Denver Metropolitan Area. *J. Wildl. Manage.* 77, 297–305.<br><br>Poessel, S. A., Gese, E. M. & Young, J. K. (2017). Environmental factors influencing the occurrence of coyotes and conflicts in urban areas. Landsc. *Urban Plan.* 157, 259–269.<br><br>Timm, R. M., Baker, R. O., Bennett, J. R. & Coolahan, C. C. (2004). Coyote Attacks: An Increasing Suburban Problem. *Trans. North Am. Wildl. Nat. Resour. Conf.* 69, 67–88.<br><br>Timm, R. M., Baker, R. O. (2007). A history of urban coyote problems. *Wildl. Damage Manag. Conf. Proc.*<br><br>White, L. A., Gehrt, S. D. (2009). Coyote attacks on humans in the United States and Canada. *Hum. Dimens. Wildl.* 14, 419–432.<br><br>Young, S., Malpeli, K. (2015). Coyote Ecology and Conflicts with Humans across the Urban-Wildland Gradient: Identifying the Potential Impacts of Changing Land Use. 1–15.
	Wolf<br><br>*Canis lupus*	Behdarvand N., Kaboli M., Ahmadi M. et al. (2014). Spatial risk model and mitigation implications for wolf–human conflict in a highly modified agroecosystem in western Iran. *Biological Conservation* 177:156-164.<br><br>Behdarvand N., Kaboli M. (2015). Characteristics of Gray Wolf Attacks on Humans in an Altered Landscape in the West of Iran. *Human Dimensions of Wildlife* 20:112–122.<br><br>Butler L., Dale B., Beckmen K., Farley S. (2011). Findings Related to the March 2010 Fatal Wolf Attack near Chignik Lake, Alaska. Wildlife Special Publication, ADF&G/DWC/WSP-2011-2. Palmer, Alaska.<br><br>Hindrikson M., Möls M., Valdmann H. (2017). The patterns of wolf attacks on humans: an example from the 19th century European Russia. *Baltic Forestry* 23(2):432-437.<br><br>Jhala, Y. V. & Sharma, D. K. (1997). Child Lifting by Wolf in Eastern Uttar Pradesh, India. *J. Wildl.*

		Res. 94–101.<br><br>Jhala, Y. V. (2003). Status, ecology and conservation of the Indian wolf. *J. Bombay Nat. Hist. Soc.* 2–3.<br><br>Linnell J.D.C., Andersen R., Andersone, Z. et al. (2002). The fear of wolves: A review of wolf attacks on humans. NINA - Oppdragsmeld 731, 1–65.<br><br>Linnell J.D.C., Kovtun E., Rouart I. (2021). Wolf attacks on humans: an update for 2002–2020. NINA Report 1944 Norwegian Institute for Nature Research.<br><br>McNay M.E. (2002). Wolf-human interactions in Alaska and Canada: a review of the case history. *Wildlife Society Bulletin* 30(3):831-843.<br><br>Nowak S., Szewczyk M., Tomczak P. et al. (2021). Social and environmental factors influencing contemporary cases of wolf aggression towards people in Poland. *European Journal of Wildlife Research* 67:69.<br><br>Rajpurohit K.S. (1999). Child lifting: wolves in Hazaribagh, India. *Ambio* 28:162-166.
Bibliography on more than two large carnivore species		Acharya K. P., Paudel P. K., Neupane P. R., Köhl, M. (2016). Human–wildlife conflicts in Nepal: patterns of human fatalities and injuries caused by large mammals. *PLoS ONE* 11:1-18.<br><br>Bombieri, G., Delgado, M.M., Russo, L.F. et al. (2018). Patterns of wild carnivore attacks on humans in urban areas. *Scientific Reports* 8: 17728.<br><br>Conover, M. R. Numbers of human fatalities, injuries, and illnesses in the United States due to wildlife. Human–Wildlife Interact. 13, 264–276 (2019).<br><br>Floyd T. (1999). Bear-inflicted human injury and fatality. *Wilderness and Environmental Medicine* 10: 75-87.<br><br>Garrote, P.J., Delgado, M.M., López-Bao, J.V. et al. (2017). Individual attributes and party affect large carnivore attacks on humans. *European Journal of Wildlife Research* 63: 80.<br><br>Lamichhane B.R., Persoon G.A., Leirs H. et al. (2018). Spatio-temporal patterns of attacks on human and economic losses from wildlife in Chitwan National Park, Nepal. *PLoS ONE* 13(4): e0195373.<br><br>Mukeka J.M., Ogutu J.O., Kanga E., Røskaft E. (2019). Human-wildlife conflicts and their correlates in Narok County, Kenya. *Global Ecology and Conservation* 18: e00620.<br><br>Nabi D.G., Tak S.R., Kangoo K.A., Halwai, M. A. (2009). Increasing incidence of injuries and fatalities inflicted by wild animals in Kashmir. *Injury* 40: 87–89.<br><br>Packer C., Shivakumar S., Athreya V. et al. (2019). Species-specific spatiotemporal patterns of leopard, lion and tiger attacks on humans. *J Appl Ecol* 56:585– 593.<br><br>Penteriani V., Delgado M.M., Pinchera F. et al. (2016). Human behaviour can trigger large carnivore attacks in developed countries. *Scientific Reports* 6: 20552.<br><br>Penteriani V., Bombieri G., Fedriani J. M. et al. (2017). Humans as prey: coping with large carnivore attacks using a predator–prey interaction perspective. *Human–Wildlife Interactions* 11:192–207.<br><br>Penteriani V., Bombieri G., Delgado M.M. et al. (2020). Patterns of Bear Attacks on Humans, Factors Triggering Risky Scenarios, and How to Reduce Them. In *Bears of the World: Ecology, Conservation and Management* (eds. Penteriani, V. & Melletti, M.) 239–249 (Cambridge University Press, UK).<br><br>Silwal T., Kolejka J., Bhatta B.P. et al. (2017). When, where and whom: assessing wildlife attacks on people in Chitwan National Park, Nepal. *Oryx* 51:370–377.

	Smith, T. S., Herrero, S. (2018). Human–bear conflict in Alaska: 1880–2015. *Wildlife Society Bulletin* 42:254–263.
